# Evaluation of large language models in generating and optimizing educational materials for neonatal home oxygen therapy

**DOI:** 10.3389/frai.2026.1770564

**Published:** 2026-03-27

**Authors:** Zhendong Liu, Xiaoping Yang, Yu Zhang, Yujing Xu, Yue Xiang, Hongyan Wang

**Affiliations:** 1 School of Nursing, Gansu University of Chinese Medicine, Lanzhou, China; 2 Department of Neonatology, Gansu Provincial Maternal and Child Health Hospital, Lanzhou, China

**Keywords:** bronchopulmonary dysplasia, health education materials, large language models, neonatal home oxygen therapy, patient education, prompt engineering, readability

## Abstract

**Background:**

Neonatal Home Oxygen Therapy (NHOT) is a critical treatment for premature infants with Bronchopulmonary Dysplasia (BPD). However, existing health education materials are generally difficult to read, particularly for grandparent caregivers with lower educational backgrounds. This study aimed to systematically evaluate the capacity of six major Large Language Models (LLMs) to generate and optimize NHOT health education materials.

**Methods:**

Six LLMs were included: ChatGPT-5.1, Claude 4.5 Sonnet, Gemini 2.5 Pro, Grok-4.1, Qwen-3-Max, and DeepSeek-V3.2. Each model generated 20 texts under three prompting strategies—baseline (Prompt A), simplification (Prompt B), and rewriting (Prompt C)—yielding 360 texts in total. Twenty WeChat public health articles served as the human-authored baseline. Subjective evaluation employed C-DISCERN, C-PEMAT (understandability and actionability), and a medical accuracy Likert scale, supplemented by objective linguistic analysis using the Alpha Readability Chinese (ARC) tool.

**Results:**

All models demonstrated superior medical accuracy compared to the human baseline (Likert median 1.0, against 2.0 for the original articles). Under baseline conditions, Qwen achieved the highest content quality (C-DISCERN median 57.0), while Claude attained perfect actionability scores. The simplification prompt (Prompt B) significantly reduced C-DISCERN scores across all models (all *p* < 0.001) without meaningfully improving understandability or actionability. In the rewriting task (Prompt C), all models significantly enhanced the understandability of original texts (*p* < 0.01), with Grok and Qwen additionally improving content quality and actionability. Linguistic analysis revealed that prompt optimization improved semantic accuracy and reduced semantic noise, but at the cost of decreased lexical richness.

**Conclusion:**

LLMs demonstrate significant potential for optimizing existing health education materials, performing more reliably in rewriting mode than in *de novo* generation. Simplistic “plain language” instructions risk compromising content quality, highlighting the need for carefully designed prompts that balance accuracy, clarity, and completeness. All AI-generated materials require rigorous review by qualified clinical professionals prior to distribution.

## 1 Introduction

Bronchopulmonary Dysplasia (BPD) is the most common chronic lung disease in premature babies. It affects up to 50% of infants with very low birth weight (Bancalari and Jain, 2019). For babies with BPD, Home Oxygen Therapy (HOT) has become a key treatment. It helps prevent harm from low oxygen levels, lowers the risk of high blood pressure in the lungs, helps the baby grow, and allows them to leave the hospital sooner (Kotecha and Allen, 2002; Ellsbury et al., 2004). Studies show that home oxygen therapy can significantly improve the baby’s weight gain and brain development (Baraldi et al., 1997; Groothuis and Rosenberg, 1987).

However, doing home oxygen therapy has many challenges. In the US, the number of BPD babies using oxygen at home varies a lot between hospitals, ranging from 1 to 37%, and even reaching 95% in some centers (Lagatta et al., 2020). In China, there are also big differences between regions. This is often linked to the economic level of each province, and there are no unified guidelines based on evidence (Jiang et al., 2023). Also, parents are often very anxious about taking their baby home with oxygen. This anxiety is highest when they leave the hospital and slowly goes down as the baby breathes better (Zanardo and Freato, 2001). Therefore, we really need high-quality, easy-to-understand education materials. This is essential to help parents feel confident and ensure home oxygen therapy is done safely.

Health Literacy means a person’s ability to find, understand, judge, and use health information to make decisions (World Health Organization, 2025). It plays a key role in preventing and controlling disease. Low health literacy is linked to higher hospital admission rates, worse self-care, missing medicine doses, and higher medical costs (Berkman et al., 2011). In China, health literacy levels are still quite low. A 2019 report showed that only about 19.17% of people aged 15–69 have basic health literacy. There is a clear gap between cities and rural areas (24.81% vs. 15.67%) and between different regions (Li et al., 2022; Yang et al., 2020).

The readability of patient education materials is a big factor in health literacy. Reviews show that most patient materials are harder to read than the recommended 6th to 8th-grade level. This problem did not get better between 2001 and 2022 (Okuhara et al., 2025). This issue is very obvious in China. A study on materials for Lupus patients found they were hard to read and needed simpler words and shorter sentences (Wang et al., 2020). Caregivers for newborns on home oxygen often include grandparents. They may have lower education levels and find medical terms or complex instructions confusing. This makes it urgent to develop simple and clear health education materials.

Recently, Large Language Models (LLMs) like ChatGPT, Claude, and Gemini have shown great potential in healthcare. These models use deep learning to understand complex medical ideas and write high-quality text (Achiam et al., 2023). LLMs are used in many areas, including helping doctors make decisions, teaching medicine, talking to patients, and writing medical notes (Thirunavukarasu et al., 2023; Singhal et al., 2023).

LLMs are especially good at patient education. Several studies checked how accurate and readable ChatGPT and GPT-4 were when answering common medical questions. The results showed their answers were as accurate as human experts and sometimes easier to understand (Jo et al., 2024; Chen et al., 2024; Lee et al., 2023). Research in fields like bone surgery, cancer, and heart health confirmed that LLMs can create high-quality patient materials and make them much easier to read (Nian et al., 2025; Gordon et al., 2024; Samaan et al., 2023). A review of 17 studies showed that LLMs have significant potential to make health information easier to access and understand (Aydin et al., 2024).

More importantly, studies prove that LLMs can lower the reading difficulty of existing medical texts. Research showed that using simple prompts with ChatGPT improved patient manuals significantly (Swisher et al., 2024). Another study found that ChatGPT-4.0 and Google Gemini were moderately effective at making pediatric bone surgery materials easier to read in both English and Spanish (Nian et al., 2025). These findings suggest that LLMs could be a powerful tool to improve current health education materials and help people with low health literacy.

Although LLMs show great promise, there are still challenges in healthcare. “Prompt Engineering” (how we design the questions we ask the AI) is a key factor affecting quality (White, 2023). Studies show that small changes in the prompt can lead to very different answers, and different models react differently (Wang et al., 2024). In medicine, designing prompts is common, but creating prompts that ensure both medical accuracy and easy reading is still an open question (Zaghir et al., 2024).

Also, “hallucinations” (when AI makes things up) are a big concern. One study found that models had a 1.47% hallucination rate in clinical notes (Asgari et al., 2025). In medical summaries, hallucinations were found in almost all cases, mostly regarding symptoms, diagnosis, or medicine (Roustan and Bastardot, 2025). Large language models basically predict statistics and do not care about real-world facts. Their output should not be treated the same as human reasoning based on evidence. This is especially important in medicine, where accuracy and responsibility are vital (Bélisle-Pipon, 2024). Therefore, strict checking and quality assessment are essential for the safe use of LLMs in health education.

Developing Chinese medical materials faces unique challenges. Chinese is very different from English in structure, vocabulary, and expression. This means we need special tools to assess and improve Chinese materials. The Chinese version of the Patient Education Materials Assessment Tool (C-PEMAT) has been proven effective and reliable for checking how easy it is to understand and use Chinese materials (Shan et al., 2023a). Also, tools for assessing Chinese health literacy are constantly improving (Li and Liu, 2020).

Research on education for people with low health literacy suggests that using videos, oral communication, and less text can effectively improve results (Li et al., 2023). In poor areas of China, using a mix of media methods is more effective than just one strategy (Li et al., 2023). These findings give us important references for using LLMs to improve Chinese health education materials.

While there is growing research on LLMs for English medical materials, there is a lack of systematic assessment for Chinese, especially in newborn care. Home oxygen therapy is a complex medical task. Parents need to master many skills, like using oxygen equipment, checking blood oxygen levels, and assessing the baby’s condition (Kapur et al., 2020). In China, grandparents often share caregiving duties, but they often have lower education and limited health literacy. This creates an urgent need for targeted, easy-to-understand education materials.

Therefore, this study aims to systematically assess the ability of major Large Language Models to generate and improve health education materials for neonatal home oxygen therapy. The specific goals are: To compare the quality of materials generated by six representative LLMs (ChatGPT, Claude, Gemini, Grok, Qwen, DeepSeek) under baseline conditions; To assess how different prompt strategies (baseline, simplification, rewriting) affect the quality and readability of the content; To verify the ability of LLMs to optimize existing online science materials through rewriting tasks; To fully evaluate the quality and features of the content using a mix of subjective assessment (DISCERN, C-PEMAT, medical accuracy) and objective analysis (Alpha Readability Chinese).

The innovation of this study is that it is the first to systematically compare multiple major LLMs for specialist health education materials in the Chinese context. It is the first to use objective semantic analysis tools to evaluate the linguistic features of Chinese medical materials. It is also the first to explore the potential of LLMs specifically for neonatal home oxygen therapy. The results will provide important scientific evidence for using AI to improve Chinese health education materials and improve information access for people with low health literacy.

## 2 Related work

### 2.1 LLMs for patient education material generation and evaluation

A growing body of literature has examined the use of LLMs to generate and improve patient-facing health content. Studies across multiple clinical domains have demonstrated that LLMs can produce materials with accuracy comparable to human experts while achieving higher readability scores (Chen et al., 2024; Jo et al., 2024; Lee et al., 2023). A scoping review of 17 studies confirmed the significant potential of LLMs for improving access to health information (Aydin et al., 2024). More directly relevant to the present work, Will et al. (2025) and Swisher et al. (2024) showed that LLM-revised patient materials score markedly higher on standard readability scales than their originals, while Nian et al. (2025) found moderate effectiveness in a multilingual pediatric orthopedic setting. However, most existing studies focus on English-language materials; systematic evaluation of LLMs for Chinese-language health education—particularly in specialized neonatal contexts—remains scarce, a gap the present study addresses.

### 2.2 Prompt engineering for medical text simplification

Prompt engineering has emerged as a critical determinant of LLM output quality. A scoping review identified prompt design as the most frequently applied methodology in medical AI studies, yet noted the absence of standardized best practices (Zaghir et al., 2024). Wang et al. (2024) demonstrated that minor prompt variations can produce substantially different outputs across models. In patient education specifically, Ellison et al. (2025) showed that prompt design was the key driver of readability improvements, but that naively worded simplification prompts could inadvertently reduce content quality—a finding directly echoed in our Prompt A versus Prompt B comparison. Mishra et al. (2024) similarly emphasized that prompt optimization must balance language simplicity with professional completeness, and called for standardized output evaluation frameworks.

### 2.3 Sequential prompting and retrieval-augmented generation for clinical text

Two important technical advances directly inform the limitations and future directions of the present study. Dehkordi et al. (2024) introduced a sequential prompting approach for simplifying electronic health records (EHRs), in which a two-step strategy—first summarizing key content, then converting it into patient-accessible language—achieved higher accuracy and lower hallucination rates than single-step prompting. This finding is relevant to our Prompt C design: providing LLMs with structured inputs may yield more faithful simplifications than processing raw online articles. Building on this, Dehkordi et al. (2025) proposed a retrieval-augmented generation (RAG) framework for discharge note comprehensibility, demonstrating that dynamically retrieved domain knowledge can improve both correctness and completeness in ways unattainable through prompt engineering alone. Both studies highlight the information completeness problem—a limitation we acknowledge in our evaluation framework and identify as a key direction for future work.

### 2.4 Evaluation frameworks for Chinese health education materials

The evaluation of Chinese-language health materials requires dedicated instruments. Shan et al. (2023a) and Shan et al. (2023b) translated and validated both the C-DISCERN and C-PEMAT tools, establishing reliable measures of information quality, understandability, and actionability in the Chinese context. Prior work has shown that Chinese patient education materials frequently exceed recommended reading difficulty levels (Wang et al., 2020). The present study extends this evaluation tradition by additionally incorporating the Alpha Readability Chinese (ARC) tool (Lei et al., 2024) for objective linguistic feature analysis—quantifying lexical richness, syntactic complexity, semantic noise, and semantic accuracy—enabling a more complete characterization of LLM output than subjective ratings alone can provide.

## 3 Methods

### 3.1 Study design

We used a cross-sectional comparative design for this study. Our goal was to evaluate how well Large Language Models (LLMs) can create and improve health education materials for Neonatal Home Oxygen Therapy (NHOT). The study had three stages: (1) Baseline generation: testing the quality of text generated by LLMs without special instructions; (2) Prompt optimization: testing if specific prompts designed to lower reading difficulty work effectively; and (3) Rewriting: testing if LLMs can turn existing online science articles into easy-to-read materials.

### 3.2 Selection of LLMs

We chose six representatives general LLMs. This list includes top international models and Chinese-optimized models to get a full picture of their performance in Chinese medical contexts:

International models: ChatGPT-5.1 (OpenAI), Claude 4.5 Sonnet (Anthropic), Gemini 2.5 Pro (Google), Grok-4.1 (xAI).

Domestic Chinese models: Qwen-3-Max (Alibaba Cloud), DeepSeek-V3.2.

All six LLMs (ChatGPT-5.1, Claude 4.5 Sonnet, Gemini 2.5 Pro, Grok-4.1, Qwen-3-Max, and DeepSeek-V3.2) were accessed exclusively through their official, public-facing web interfaces rather than via application programming interfaces (APIs). To accurately simulate the real-world usage behavior of general family caregivers—who typically do not adjust technical settings—all models were operated using their default generation parameters (e.g., default temperature and top-p). Specifically, for models offering dynamic processing duration options, ChatGPT-5.1 and Grok-4.1 were explicitly set to their “auto” modes. Advanced custom instructions or manual parameter adjustments were not utilized, ensuring the models were evaluated in their completely standard, default forms.

All models were accessed via their official websites in November 2025.

### 3.3 Data sources and baseline

To set a baseline for evaluation and obtain material for the rewriting task, we searched WeChat, China’s largest social media platform. We conducted the search using the exact keywords: “neonatal home oxygen therapy” and “home oxygen for premature infants.” No specific date range restrictions were applied to the search.

The initial relevance ranking of the search results was automatically determined by WeChat’s default comprehensive search algorithm. We sequentially screened the top results to select 20 articles intended for patients or family caregivers.

Inclusion criteria: (1) The primary topic focused on home oxygen care for newborns/infants; (2) The content was predominantly text-based; (3) The publisher accounts were strictly limited to verified official accounts of hospital departments, individual doctors and professional health media.

Exclusion criteria: (1) Purely video-based content; (2) Commercial advertisements; (3) Academic papers or expert consensus documents; (4) Articles published by personal blogs, or other unverified accounts.

Selection process: Two authors (ZL and XY) independently conducted the extraction and screening process based on the criteria above. Any disagreements regarding article inclusion were resolved through a consensus discussion. If an agreement could not be reached, a third senior author (HW) acted as an adjudicator to make the final decision.

### 3.4 Prompt engineering

We designed three different prompt strategies. Each model generated 20 texts for each strategy independently (totaling 6 × 20 × 3 = 360 samples). We cleared the chat history after each generation to avoid interference from previous context.

Prompt development and pilot testing: The three prompt strategies (A, B, and C) were developed through an iterative process involving clinical experts. Initially, the research team drafted candidate prompts based on common clinical inquiries. A pilot test was then conducted where each LLM generated a small subset of 5 sample texts per prompt. These intermediate results were reviewed by the authors to evaluate whether the prompts effectively steered the models toward the desired structural and linguistic outputs. Based on this expert feedback, Prompt B was explicitly refined to include the constraints “avoid professional terms” and “use short, simple sentences” to ensure a strict simplification effect. Once validated in the pilot phase, the finalized prompts were applied for the main generation task.

Prompt A (baseline strategy): Mimics a question from a typical parent. Instruction: “Please write an educational material about neonatal home oxygen therapy for family caregivers (including parents and grandparents) so they can understand it easily.”

Prompt B (simplification strategy): Designed to test the model’s ability to adapt to people with low health literacy (especially older caregivers). The instruction added specific rules: “Please write an educational material about neonatal home oxygen therapy for family caregivers (including grandparents with lower education). Please be sure to use the most everyday words (avoid professional terms) and use short, simple sentences to ensure the reading difficulty is as low as possible.”

Prompt C (rewriting strategy): Evaluates the ability to improve existing materials. Instruction: “Please rewrite the following text about neonatal home oxygen therapy. Use everyday words and simple short sentences to make it suitable for grandparents with lower education levels: [Insert original online text].”

### 3.5 Evaluation metrics

We used a mix of subjective human assessment and objective semantic analysis.

#### 3.5.1 Content quality and applicability

Two independent reviewers (YZ and HW) graded all generated texts blindly. Both reviewers are specialist Neonatal Intensive Care Unit (NICU) nurses with over 7 years of clinical experience, and both have served as NICU head nurses.

To ensure evaluation reliability, we assessed inter-rater agreement based on the raw scoring data. The Intra-class Correlation Coefficient (ICC) was calculated for the continuous variables (C-DISCERN scores and C-PEMAT Actionability and Understandability scores), while Cohen’s Kappa was utilized for the categorical medical accuracy Likert scores.

Minor scoring discrepancies between the two reviewers were resolved by calculating the average score and rounding it to the nearest whole number. However, recognizing the different scoring ranges of the evaluation tools, we established specific thresholds to define “significant disagreements” that required a strict adjudication mechanism: a difference of >1 point on the medical accuracy Likert scale, >5 points on the total C-DISCERN score, or >10% on the C-PEMAT score. When a discrepancy exceeded these predefined thresholds, the two reviewers first engaged in a consensus discussion. If an agreement could not be reached, other members of the research team were involved in a joint discussion to make the final, collective decision.

Quality assessment: We used the Chinese version of the DISCERN tool (C-DISCERN) (Shan et al., 2023b). It has 16 items (scored 1–5) to judge the reliability of the information, how treatment choices are described, and overall quality. Although the DISCERN instrument was originally designed to evaluate patient materials regarding treatment options, it has been widely adapted in recent literature to assess the overall reliability and quality of general health education materials. For procedural content like NHOT, we focused predominantly on items evaluating the clarity of information, the description of risks (e.g., equipment failure or oxygen toxicity), and the overall reliability of the content (Uzun, 2023; Alan and Alan, 2023).

Understandability and actionability: We used the Chinese Patient Education Materials Assessment Tool (C-PEMAT) (Shan et al., 2023a). This covers Understandability (17 items) and Actionability (7 items). The final score is a percentage (%).

Medical accuracy: We used a 5-point Likert scale to check for medical fact errors and hallucinations (1 = completely accurate, 5 = severe errors that endanger life) (Jebb et al., 2021).

#### 3.5.2 Linguistic feature analysis

To objectively measure the language features of the text, we used the Alpha Readability Chinese (ARC) tool (Lei et al., 2024). The indicators included:

Lexical richness: Measures how diverse the vocabulary is.

Syntactic richness: Measures how complex the sentence structures are.

Semantic noise: Measures the amount of redundant or distracting information.

Semantic accuracy: Divided into Noun (N) and Verb (V) accuracy, measuring how precise the word choice is.

### 3.6 Statistical analysis

We used R software (version 4.5.0) operating within the RStudio interface for data analysis. Normality was checked using the Shapiro–Wilk test. Since most scores (DISCERN, Likert) and some Linguistic Feature Analysis metrics did not follow a normal distribution, we mainly used non-parametric tests.

Multiple group comparison: We used the Kruskal-Wallis H test to compare differences between the six models. If significant differences were found, we used the Mann–Whitney U test with Bonferroni correction for pairwise comparisons.

Within-group comparison (Prompt A vs. B): We used the Mann–Whitney U test to analyze differences within the same model under different prompt strategies.

Paired comparison (Original vs. Prompt C): We used the Wilcoxon Signed-Rank Test to compare the original texts with the AI-rewritten texts. All tests were two-sided, and a *p*-value < 0.05 was considered statistically significant.

## 4 Results

This study included 20 original WeChat science articles about neonatal home oxygen therapy as a baseline control. We evaluated 360 texts generated by six Large Language Models (ChatGPT, Claude, DeepSeek, Gemini, Grok, Qwen) using three different prompt strategies (Prompt A, B, C). The evaluation covered content quality (DISCERN), suitability (PEMAT, including understandability and actionability), and medical accuracy (Likert score).

The intraclass correlation coefficient (ICC) was calculated to assess inter-rater reliability for continuous variables. The ICC values were 0.89 for the C-DISCERN scores, 0.72 for the C-PEMAT Actionability scores, and 0.78 for the C-PEMAT Understandability scores, indicating substantial to excellent agreement. For the categorical medical accuracy Likert ratings, inter-rater agreement was evaluated using Cohen’s kappa, which yielded a coefficient of 0.85, demonstrating strong consistency between raters.

### 4.1 Overall performance of LLMs across prompt strategies

Table 1 presents the median and interquartile range (IQR) [Q1, Q3] for medical accuracy (Likert scale), content quality (C-DISCERN), understandability, and actionability (C-PEMAT) across all six large language models under the three prompting strategies. To establish a benchmark for evaluating the AI-generated texts, the median scores of the original human-authored WeChat articles used for the rewriting task are also included as a baseline.

**Table 1:** Summary of median evaluation scores across prompt strategies and baseline comparison.

Model	Prompt strategy	Medical accuracy (Likert)	Content quality (C-DISCERN)	Understandability (C-PEMAT, %)	Actionability (C-PEMAT, %)
WeChat original articles	Not applicable	2.0 [1.0, 3.0]	44.5 [40.0, 50.0]	83.0 [75.0, 91.8]	83.0 [83.0, 83.0]
ChatGPT-5.1	Prompt A (Baseline)	1.0 [1.0, 1.0]	49.5 [48.0, 51.2]	96.0 [92.0, 100.0]	83.0 [83.0, 100.0]
	Prompt B (Simplification)	1.0 [1.0, 1.0]	41.0 [38.8, 44.2]	100.0 [90.0, 100.0]	83.0 [83.0, 83.0]
	Prompt C (Rewriting)	1.0 [1.0, 2.0]	46.0 [41.2, 49.0]	87.0 [83.0, 94.0]	83.0 [83.0, 83.0]
Claude 4.5 Sonnet	Prompt A (Baseline)	1.0 [1.0, 1.0]	51.0 [50.0, 53.2]	100.0 [98.0, 100.0]	100.0 [100.0, 100.0]
	Prompt B (Simplification)	1.0 [1.0, 1.0]	46.5 [44.0, 48.0]	100.0 [92.0, 100.0]	100.0 [100.0, 100.0]
	Prompt C (Rewriting)	1.0 [1.0, 2.2]	43.5 [39.8, 51.0]	92.0 [89.0, 100.0]	83.0 [83.0, 88.5]
DeepSeek-V3.2	Prompt A (Baseline)	1.0 [1.0, 1.0]	47.5 [44.0, 49.2]	92.0 [83.0, 100.0]	100.0 [83.0, 100.0]
	Prompt B (Simplification)	1.0 [1.0, 1.0]	43.0 [40.0, 46.5]	92.0 [83.0, 100.0]	100.0 [83.0, 100.0]
	Prompt C (Rewriting)	1.0 [1.0, 2.0]	46.5 [42.0, 49.8]	89.5 [81.5, 96.0]	83.0 [83.0, 83.0]
Gemini 2.5 Pro	Prompt A (Baseline)	1.0 [1.0, 1.0]	49.0 [45.8, 52.0]	100.0 [92.0, 100.0]	100.0 [83.0, 100.0]
	Prompt B (Simplification)	1.0 [1.0, 1.0]	42.5 [39.8, 45.0]	100.0 [92.0, 100.0]	83.0 [83.0, 100.0]
	Prompt C (Rewriting)	1.0 [1.0, 1.0]	46.0 [42.0, 50.0]	100.0 [92.0, 100.0]	83.0 [83.0, 83.0]
Grok-4.1	Prompt A (Baseline)	1.0 [1.0, 1.0]	50.0 [48.0, 52.0]	100.0 [94.0, 100.0]	83.0 [83.0, 100.0]
	Prompt B (Simplification)	1.0 [1.0, 1.0]	46.5 [44.0, 49.2]	100.0 [100.0, 100.0]	83.0 [83.0, 95.8]
	Prompt C (Rewriting)	1.0 [1.0, 2.0]	48.5 [43.8, 51.0]	100.0 [92.0, 100.0]	83.0 [83.0, 83.0]
Qwen-3-Max	Prompt A (Baseline)	1.0 [1.0, 1.0]	57.0 [54.8, 60.2]	100.0 [100.0, 100.0]	100.0 [100.0, 100.0]
	Prompt B (Simplification)	1.0 [1.0, 1.0]	40.5 [37.8, 43.0]	100.0 [100.0, 100.0]	91.5 [83.0, 100.0]
	Prompt C (Rewriting)	1.0 [1.0, 1.0]	48.0 [43.8, 51.2]	100.0 [92.0, 100.0]	83.0 [83.0, 95.8]

Data are presented as Median [Q1, Q3]. Likert scores range from 1 to 5 (lower is better; 1 = completely accurate). C-DISCERN scores range from 16 to 80 (higher is better). C-PEMAT scores are presented as percentages (0–100%, higher is better).

Regarding medical accuracy, all AI models demonstrated high clinical safety across the three prompting strategies, consistently achieving a median Likert score of 1.0 [1.0, 1.0] (completely accurate), which outperformed the human-authored baseline (2.0 [1.0, 3.0]).

In terms of content quality (C-DISCERN), the models generally achieved their highest scores under the baseline prompt (Prompt A), with Qwen-3-Max reaching a median of 57.0. However, when executing the strict simplification instruction (Prompt B), content quality noticeably deteriorated across all models (e.g., Qwen-3-Max dropped to 40.5). When rewriting existing texts (Prompt C), the models’ quality scores largely stabilized between 43.5 and 48.5, performing comparably to or slightly better than the human-authored baseline (44.5).

For understandability (C-PEMAT), the AI models exhibited robust linguistic transformation capabilities. The scores for the generated texts overwhelmingly ranged from 90.0 to 100.0%, substantially exceeding the human baseline (83.0%). Notably, under the simplification strategy (Prompt B), almost all models achieved a perfect median understandability score of 100.0%.

Regarding actionability (C-PEMAT), the median scores for the AI-generated texts were primarily concentrated between 83.0 and 100.0%, consistently meeting or exceeding the overall baseline level (83.0%).

### 4.2 Performance differences among six models under baseline prompt (Prompt A)

**Figure 1:**
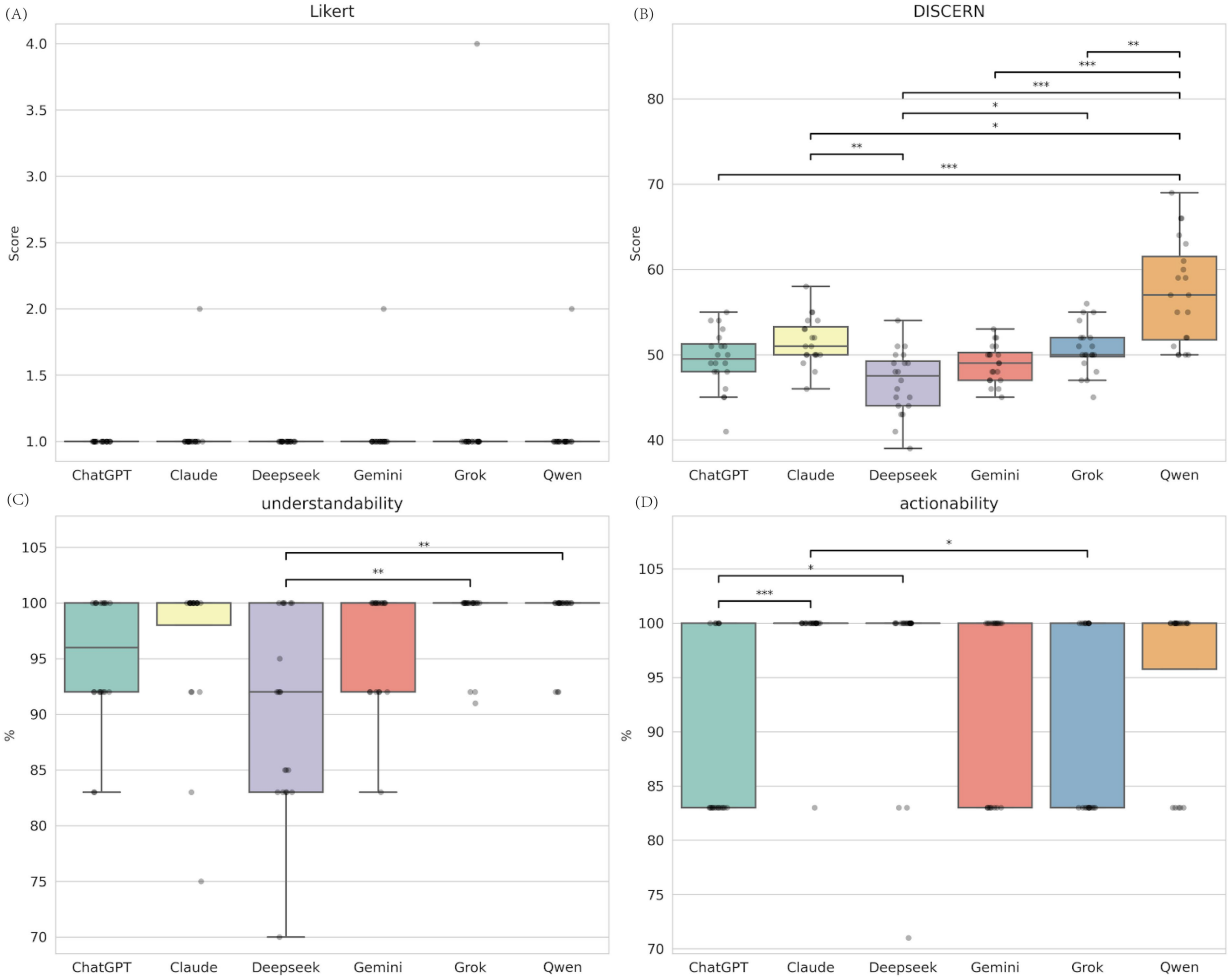
Comparative performance of six LLMs under baseline condition. **(A)** Medical accuracy assessed by the Likert scale (1 -5, lower is better); **(B)** Content quality assessed by the C-DISCERN tool (16 -80, higher is better); **(C)** Understandability score from the C-PEMAT tool (expressed as percentage, higher is better); **(D)** Actionability score from the C-PEMAT tool (expressed as percentage, higher is better). ***Indicates *p* < 0.001, **Indicates *p* < 0.01, and *Indicates *p* < 0.05.

In the baseline scenario (Prompt A), which mimics a typical parent’s question, the models performed very differently (Figure 1; Supplementary Table S1). The Kruskal-Wallis H test showed statistically significant differences in DISCERN quality scores (H(5) = 44.03, *p* < 0.001), understandability (H(5) = 23.42, *p* < 0.001), and actionability (H(5) = 25.45, *p* < 0.001). However, for medical accuracy (Likert score), the models performed similarly with no significant difference (*p* = 0.842), meaning they all provided fairly accurate medical advice.

Pairwise comparisons (Mann–Whitney U test with Bonferroni correction) showed that Qwen performed best in content quality (Md = 57.0), significantly better than ChatGPT, Claude, DeepSeek, Gemini, and Grok (all *p* < 0.05). For actionability, Claude and DeepSeek both reached full scores (Md = 100.0), with Claude significantly outperforming ChatGPT (*p* < 0.001). Although all models had high median scores for understandability, Grok and Qwen were significantly better than DeepSeek (Supplementary Tables S2–S5).

### 4.3 Performance differences among six models under simplified prompt (Prompt B)

**Figure 2:**
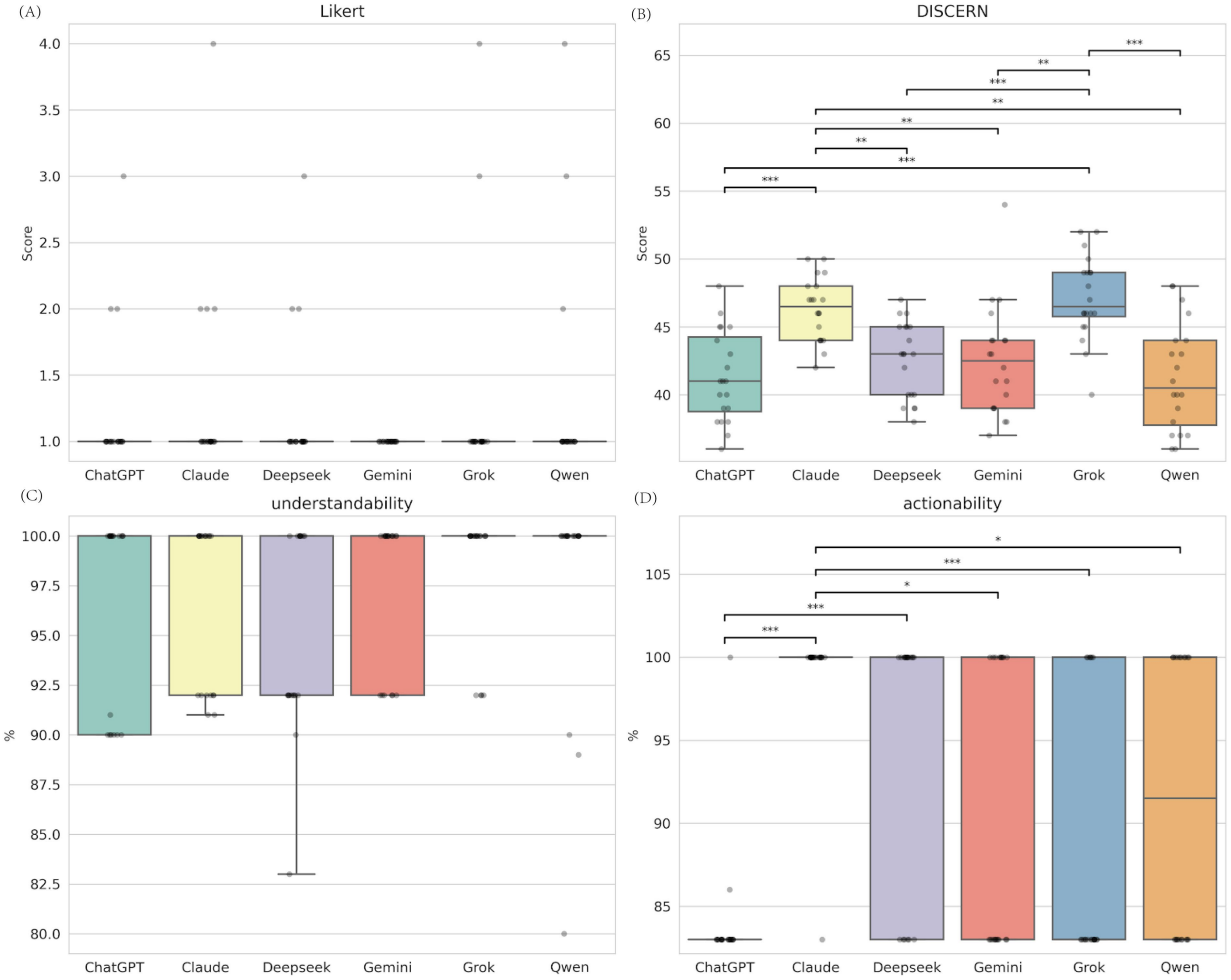
Comparative performance of six LLMs under simplification strategy. **(A)** Medical accuracy assessed by the Likert scale (1-5, lower is better); **(B)** Content quality assessed by the C-DISCERN tool (16 -80, higher is better); **(C)** Understandability score from the C-PEMAT tool (expressed as percentage, higher is better); **(D)** Actionability score from the C-PEMAT tool (expressed as percentage, higher is better).

With the Prompt B strategy, designed to create “minimalist” content, the performance gaps mainly appeared in content quality and actionability (both *p* < 0.001) (Figure 2; Supplementary Table S6). Claude and Grok tied for first place in DISCERN quality scores (Md = 46.5), significantly better than the other four models. For actionability, Claude again showed an advantage (Md = 100.0), scoring significantly higher than ChatGPT, Gemini, Grok, and Qwen (*p* < 0.05). All models achieved high levels of understandability, with no significant differences found between groups (Supplementary Tables S7–S10).

### 4.4 Performance differences among six models under rewriting task (Prompt C)

**Figure 3:**
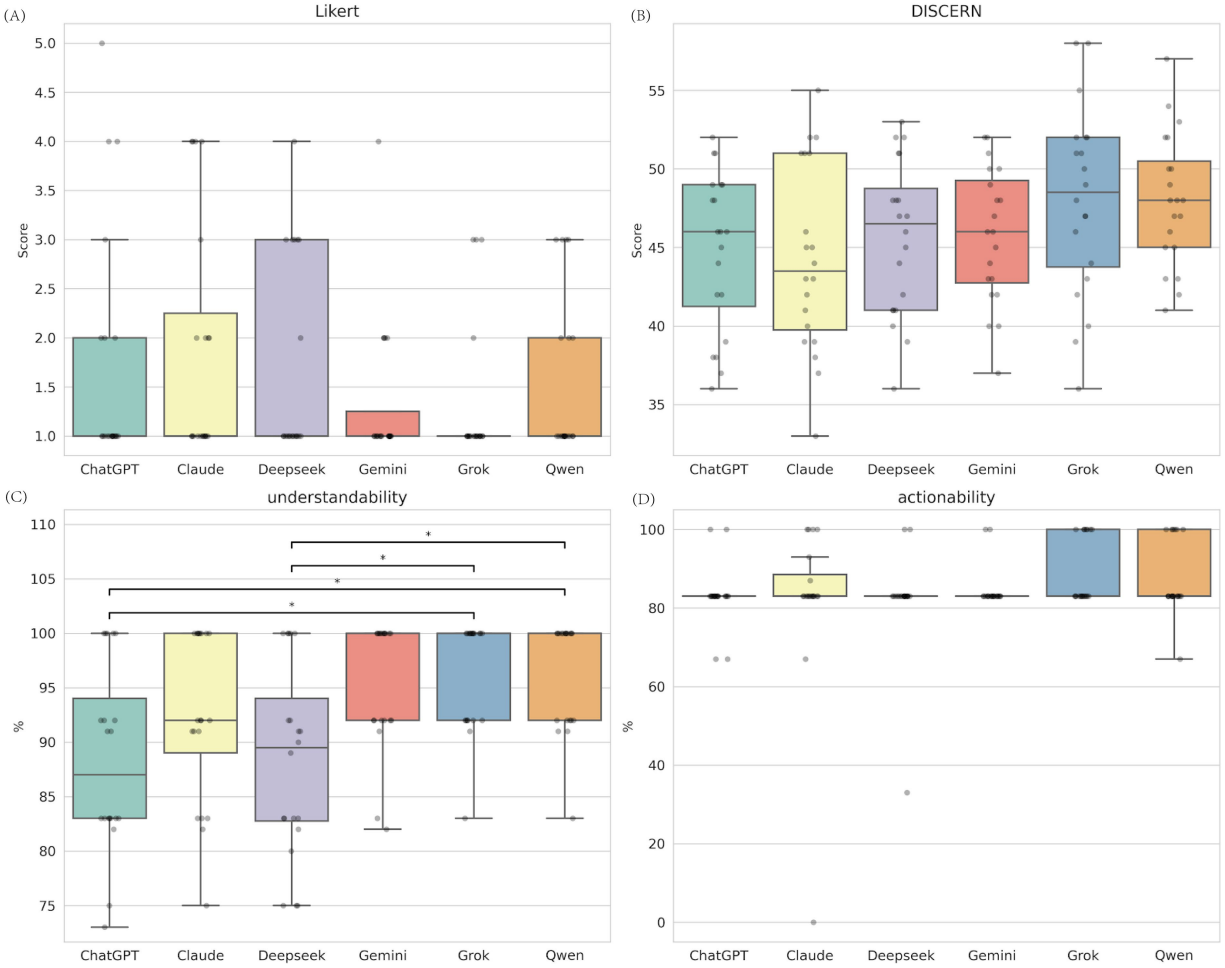
Comparative performance of six LLMs under rewriting task (Prompt C). **(A)** Medical accuracy assessed by the Likert scale (1 -5, lower is better); **(B)** Content quality assessed by the C-DISCERN tool (16 -80, higher is better); **(C)** Understandability score from the C-PEMAT tool (expressed as percentage, higher is better); **(D)** Actionability score from the C-PEMAT tool (expressed as percentage, higher is better). *Indicates *p* < 0.05.

In the task of rewriting existing texts, the overall performance of the models tended to be consistent (Figure 3; Supplementary Table S11). Significant differences were found only in understandability (H(5) = 22.79, *p* < 0.001). Grok and Qwen produced the most readable rewritten texts (Md = 100.0), significantly better than ChatGPT (Md = 87.0) and DeepSeek (Md = 89.5). There were no significant differences between models regarding content quality, actionability, or accuracy after rewriting (Supplementary Tables S12–S15).

### 4.5 Impact of prompt strategy on model performance (Prompt A vs. Prompt B)

**Figure 4:**
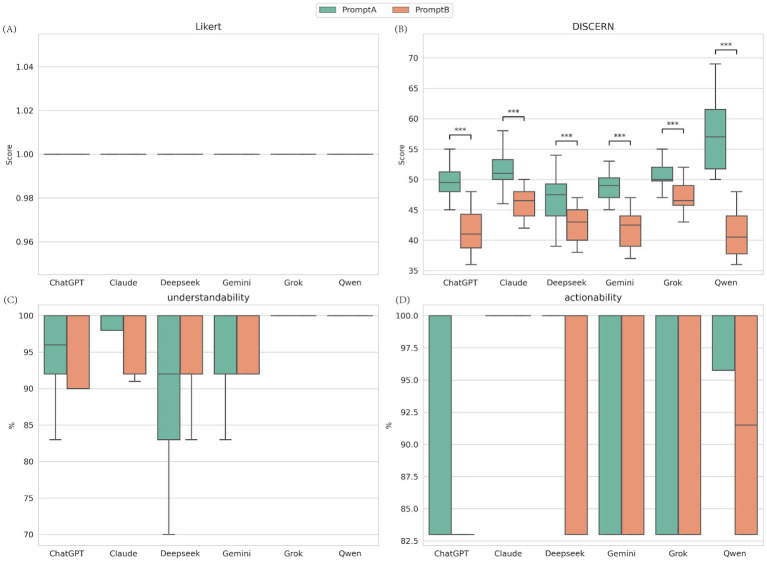
Impact of prompt strategy on quality, understandability, and actionability. **(A)** Medical accuracy assessed by the Likert scale (1 -5, lower is better); **(B)** Content quality assessed by the C-DISCERN tool (16 -80, higher is better); **(C)** Understandability score from the C-PEMAT tool (expressed as percentage, higher is better); **(D)** Actionability score from the C-PEMAT tool (expressed as percentage, higher is better). ***Indicates *p* < 0.001.

Mann–Whitney U test results showed that changing the prompt strategy had a significant negative effect on the professional quality (DISCERN) of the generated content (Figure 4; Supplementary Table S16). For all 6 models, text quality scores from Prompt A (Baseline) were significantly higher than those from Prompt B (Simplified) (all *p* < 0.001). For example, Qwen’s median DISCERN score dropped sharply from 57.0 with Prompt A to 40.5 with Prompt B. However, Prompt B, which was meant to improve readability, did not bring statistically significant improvements in understandability, actionability, or medical accuracy (*p* > 0.05).

### 4.6 Comparison of content quality between AI-rewritten and original texts (Prompt C vs. Original)

**Figure 5:**
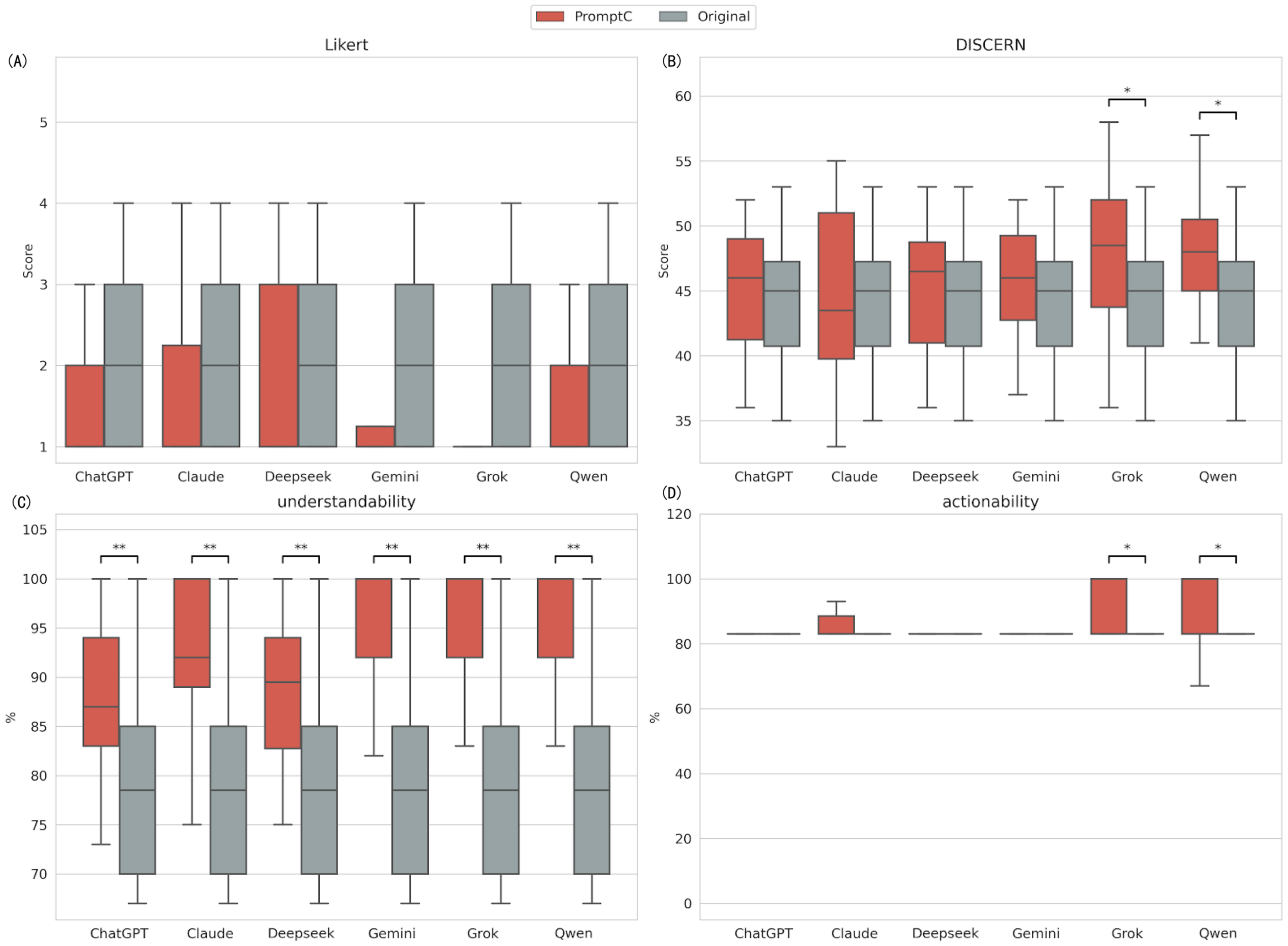
Comparison of quality and suitability between AI-rewritten and original texts. **(A)** Medical accuracy assessed by the Likert scale (1 -5, lower is better); **(B)** Content quality assessed by the C-DISCERN tool (16 -80, higher is better); **(C)** Understandability score from the C-PEMAT tool (expressed as percentage, higher is better); **(D)** Actionability score from the C-PEMAT tool (expressed as percentage, higher is better). **Indicates *p* < 0.01, and *Indicates *p* < 0.05.

Using the Wilcoxon Signed-Rank Test to compare AI-rewritten content with the original WeChat texts, the results showed that AI intervention significantly improved the quality of health education materials (Figure 5; Supplementary Table S17).

Improved understandability: The understandability of texts rewritten by all 6 models was significantly better than the original texts (*p* < 0.01). The median understandability of original texts was 78.5, while AI-rewritten texts improved to between 87.0 and 100.0.

Optimized quality and actionability: Content rewritten by Grok (*p* = 0.028) and Qwen (*p* = 0.024) had significantly higher DISCERN quality scores than the original texts. Also, Grok (*p* = 0.007) and Qwen (*p* = 0.013) scored significantly better than the original texts in actionability, offering more practical guidance.

Maintained accuracy: There was no significant difference in medical accuracy between the rewritten texts from any model and the original texts.

### 4.7 Linguistic feature differences among six models under baseline prompt (Prompt A)

**Figure 6:**
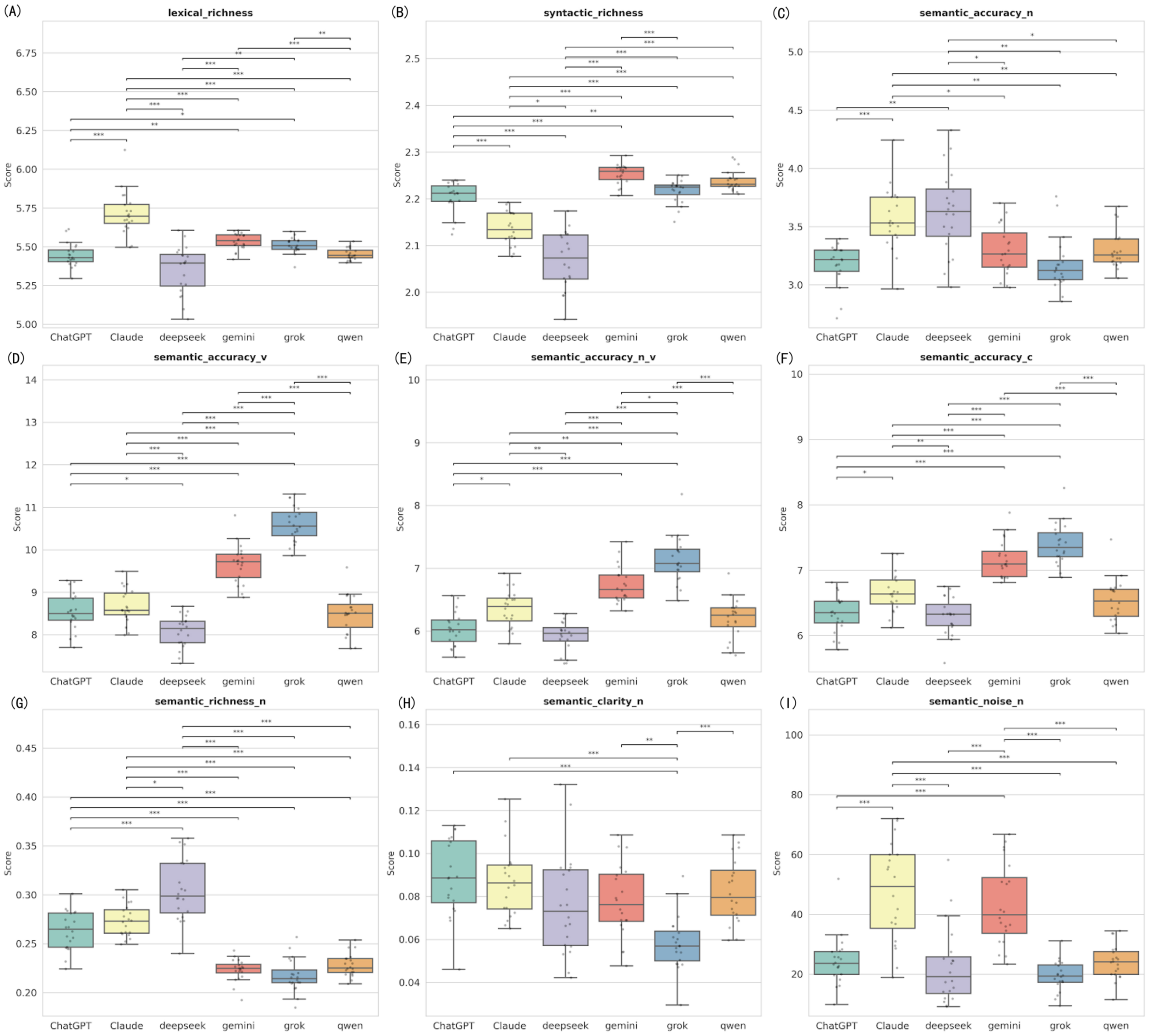
Distribution of linguistic features among six LLMs under baseline condition. The nine panels represent the nine linguistic feature indicators measured by the Alpha Readability Chinese (ARC) tool: **(A)** Lexical richness, measuring vocabulary diversity; **(B)** Syntactic richness, measuring sentence structural complexity; **(C)** Semantic accuracy for nouns (Semantic accuracy-N); **(D)** Semantic accuracy for verbs (Semantic accuracy-V); **(E)** Combined noun and verb semantic accuracy (Semantic accuracy-N_V); **(F)** Overall comprehensive semantic accuracy (Semantic accuracy-C); **(G)** Semantic richness for nouns (Semantic richness-N); **(H)** Semantic clarity for nouns (Semantic clarity-N); **(I)** Semantic noise for nouns (Semantic noise-N), measuring redundant or distracting information (lower is better). ***Indicates *p* < 0.001, **Indicates *p* < 0.01, and *Indicates *p* < 0.05.

To evaluate the underlying linguistic features of different LLMs when handling medical text, we analyzed 9 linguistic feature indicators using the Kruskal-Wallis H test. Under the baseline condition (Prompt A), the six models showed statistically significant differences in all 9 linguistic feature indicators (all *p* < 0.001) (Figure 6; Supplementary Table S18). This indicates that each model has a distinct style when generating text naturally.

Pairwise comparisons showed that Claude had the highest lexical richness (Md = 5.70) and semantic richness (Md = 0.273), significantly outperforming other models, but this came with the highest level of semantic noise (Md = 49.33). In contrast, Grok and Gemini performed excellently in accuracy indicators. Specifically for verb semantic accuracy, Grok (Md = 10.56) and Gemini (Md = 9.72) were significantly higher than ChatGPT (Md = 8.50) and DeepSeek (Md = 8.15). DeepSeek had the lowest syntactic richness (Md = 2.07) among all models, suggesting its sentence structures were the most simple (Supplementary Table S19).

### 4.8 Linguistic feature differences among six models under simplified prompt (Prompt B)

**Figure 7:**
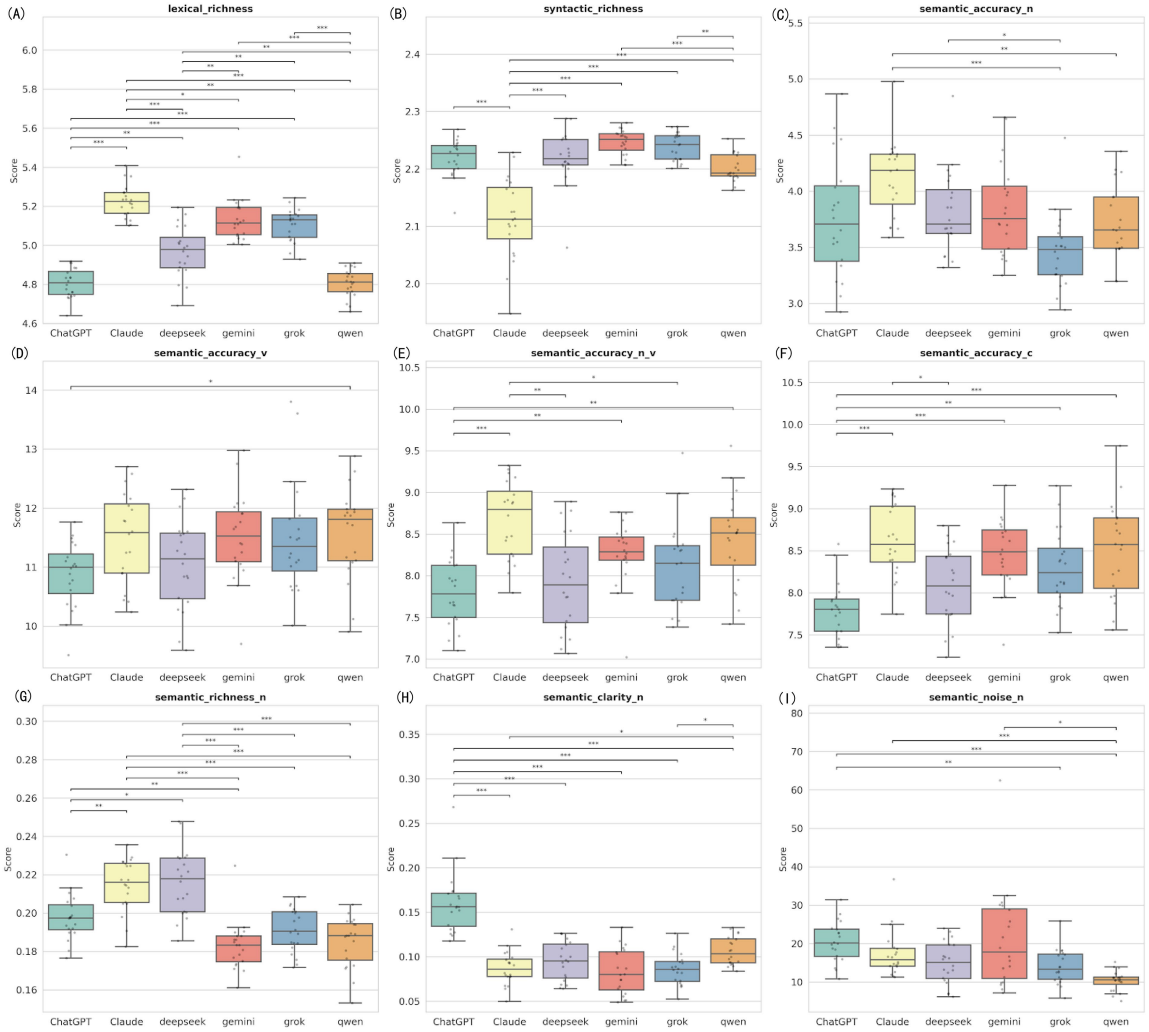
Distribution of linguistic features among six LLMs under simplification strategy. The nine panels represent the nine linguistic feature indicators measured by the Alpha Readability Chinese (ARC) tool: **(A)** Lexical richness, measuring vocabulary diversity; **(B)** Syntactic richness, measuring sentence structural complexity; **(C)** Semantic accuracy for nouns (Semantic accuracy-N); **(D)** Semantic accuracy for verbs (Semantic accuracy-V); **(E)** Combined noun and verb semantic accuracy (Semantic accuracy-N_V); **(F)** Overall comprehensive semantic accuracy (Semantic accuracy-C); **(G)** Semantic richness for nouns (Semantic richness-N); **(H)** Semantic clarity for nouns (Semantic clarity-N); **(I)** Semantic noise for nouns (Semantic noise-N), measuring redundant or distracting information (lower is better). ***Indicates *p* < 0.001, **Indicates *p* < 0.01, and *Indicates *p* < 0.05.

Under Prompt B, designed for “minimalist” content, significant linguistic feature differences remained between models (*p* < 0.05) (Figure 7; Supplementary Table S20), but the specific patterns changed. Claude continued to hold the highest lexical richness (Md = 5.22) and noun semantic accuracy (Md = 4.19). Notably, ChatGPT showed the best semantic clarity under this condition (Md = 0.156), significantly higher than all other models (*p* < 0.001), suggesting it responds best to instructions for “clear and easy to understand” text. Regarding semantic noise control, Qwen performed best (Md = 10.58), significantly lower than ChatGPT (Md = 20.15) and Gemini (Md = 17.78), showing strong ability to filter information (Supplementary Table S21).

### 4.9 Linguistic feature differences among six models under rewriting task (Prompt C)

**Figure 8:**
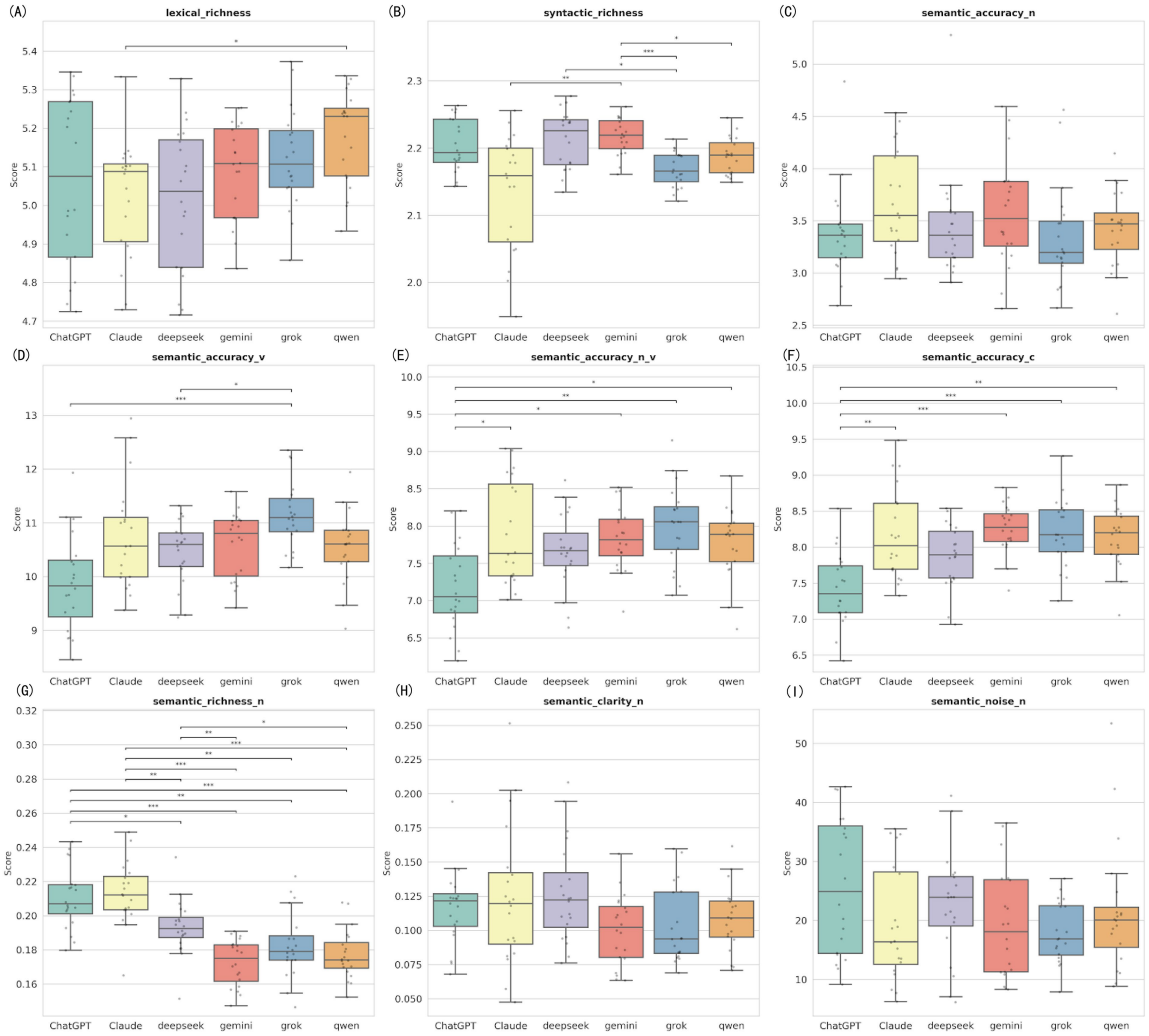
Distribution of linguistic features among six LLMs under rewriting task. The nine panels represent the nine linguistic feature indicators measured by the Alpha Readability Chinese (ARC) tool: **(A)** Lexical richness, measuring vocabulary diversity; **(B)** Syntactic richness, measuring sentence structural complexity; **(C)** Semantic accuracy for nouns (Semantic accuracy-N); **(D)** Semantic accuracy for verbs (Semantic accuracy-V); **(E)** Combined noun and verb semantic accuracy (Semantic accuracy-N_V); **(F)** Overall comprehensive semantic accuracy (Semantic accuracy-C); **(G)** Semantic richness for nouns (Semantic richness-N); **(H)** Semantic clarity for nouns (Semantic clarity-N); **(I)** Semantic noise for nouns (Semantic noise-N), measuring redundant or distracting information (lower is better). ***Indicates *p* < 0.001, **Indicates *p* < 0.01, and *Indicates *p* < 0.05.

In the rewriting task (Prompt C), the performance differences between models showed a significant trend of assimilation (Figure 8; Supplementary Table S22). While there were still statistical differences in metrics like syntactic richness and verb accuracy (*p* < 0.05), there were no longer significant differences between models in three key indicators: noun semantic accuracy (*H* = 7.61, *p* = 0.179), semantic clarity (*H* = 8.76, *p* = 0.119), and semantic noise (*H* = 7.58, *p* = 0.181). This suggests that the “rewrite” instruction tends to smooth out the unique features of different models, making their language styles more uniform. Under this condition, Grok still significantly outperformed ChatGPT (Md = 9.83) in verb accuracy (Md = 11.10) (Supplementary Table S23).

### 4.10 Impact of prompt strategy on linguistic features (Prompt A vs. Prompt B)

**Figure 9:**
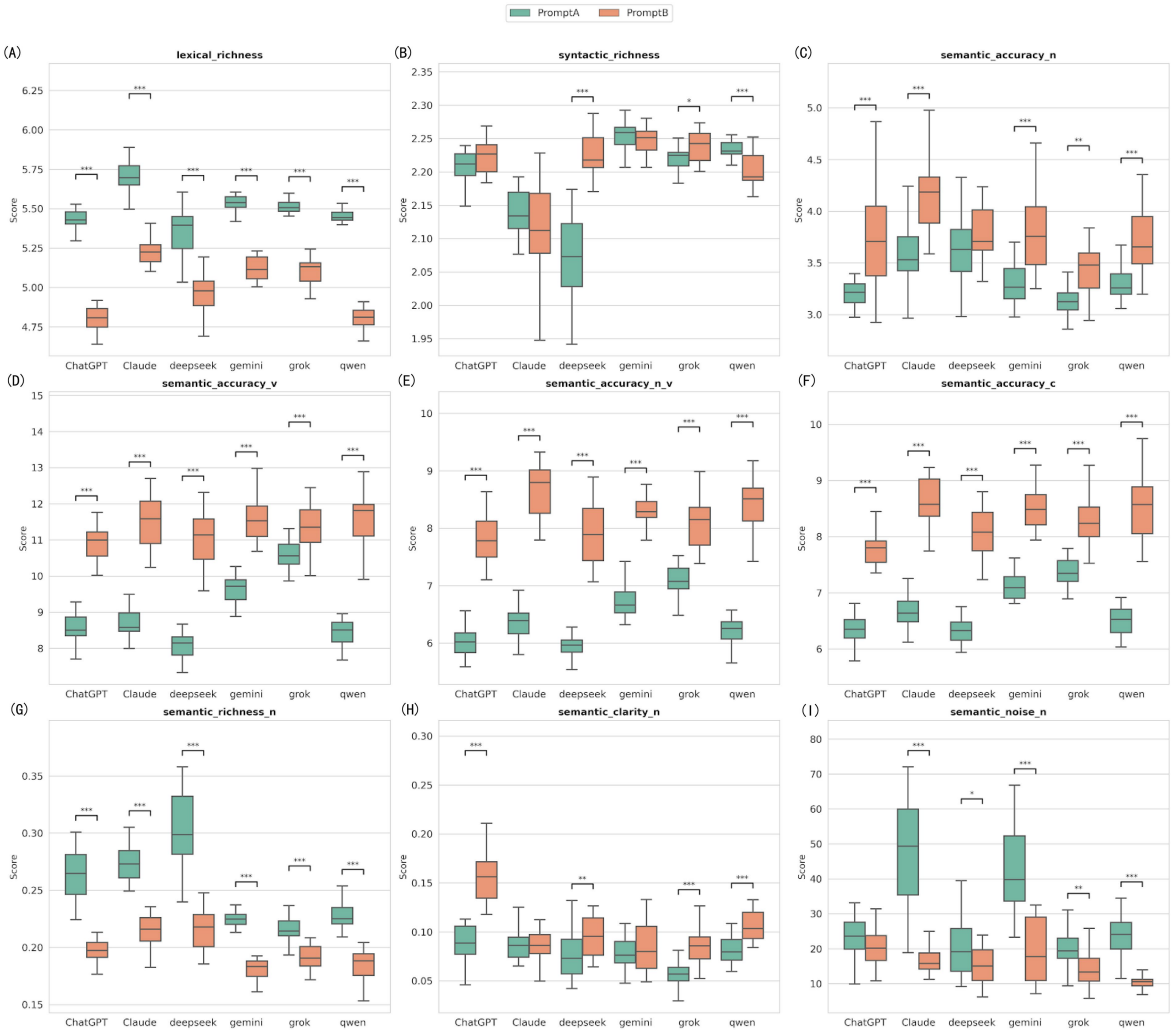
Shifts in linguistic features: comparison between baseline and simplified prompts. The nine panels represent the nine linguistic feature indicators measured by the Alpha Readability Chinese (ARC) tool: **(A)** Lexical richness, measuring vocabulary diversity; **(B)** Syntactic richness, measuring sentence structural complexity; **(C)** Semantic accuracy for nouns (Semantic accuracy-N); **(D)** Semantic accuracy for verbs (Semantic accuracy-V); **(E)** Combined noun and verb semantic accuracy (Semantic accuracy-N_V); **(F)** Overall comprehensive semantic accuracy (Semantic accuracy-C); **(G)** Semantic richness for nouns (Semantic richness-N); **(H)** Semantic clarity for nouns (Semantic clarity-N); **(I)** Semantic noise for nouns (Semantic noise-N), measuring redundant or distracting information (lower is better). ***Indicates *p* < 0.001, **Indicates *p* < 0.01, and *Indicates *p* < 0.05.

The Mann–Whitney U test revealed a consistent pattern of linguistic feature evolution for all six models when moving from the baseline prompt (Prompt A) to the optimized prompt (Prompt B) (Figure 9; Supplementary Table S24).

Improved semantic accuracy: All models showed a highly significant improvement in verb semantic accuracy (Semantic Accuracy-V) and comprehensive semantic accuracy (Semantic Accuracy-C) (*p* < 0.001). For instance, ChatGPT’s median verb accuracy rose from 8.50 to 11.00.

Suppression of semantic noise: Except for ChatGPT, semantic noise for the other five models significantly decreased under Prompt B (*p* < 0.05). Claude had the biggest drop, with its median falling sharply from 49.33 to 15.81, showing effective removal of redundant information.

Richness trade-off: Along with improved accuracy, the lexical richness and semantic richness (Semantic Richness-N) of all models significantly decreased (*p* < 0.001). This indicates that in the optimization mode, models tend to sacrifice vocabulary diversity to achieve higher accuracy and clarity.

### 4.11 Comparison of linguistic features between AI-rewritten and original texts (Prompt C vs. Original)

**Figure 10:**
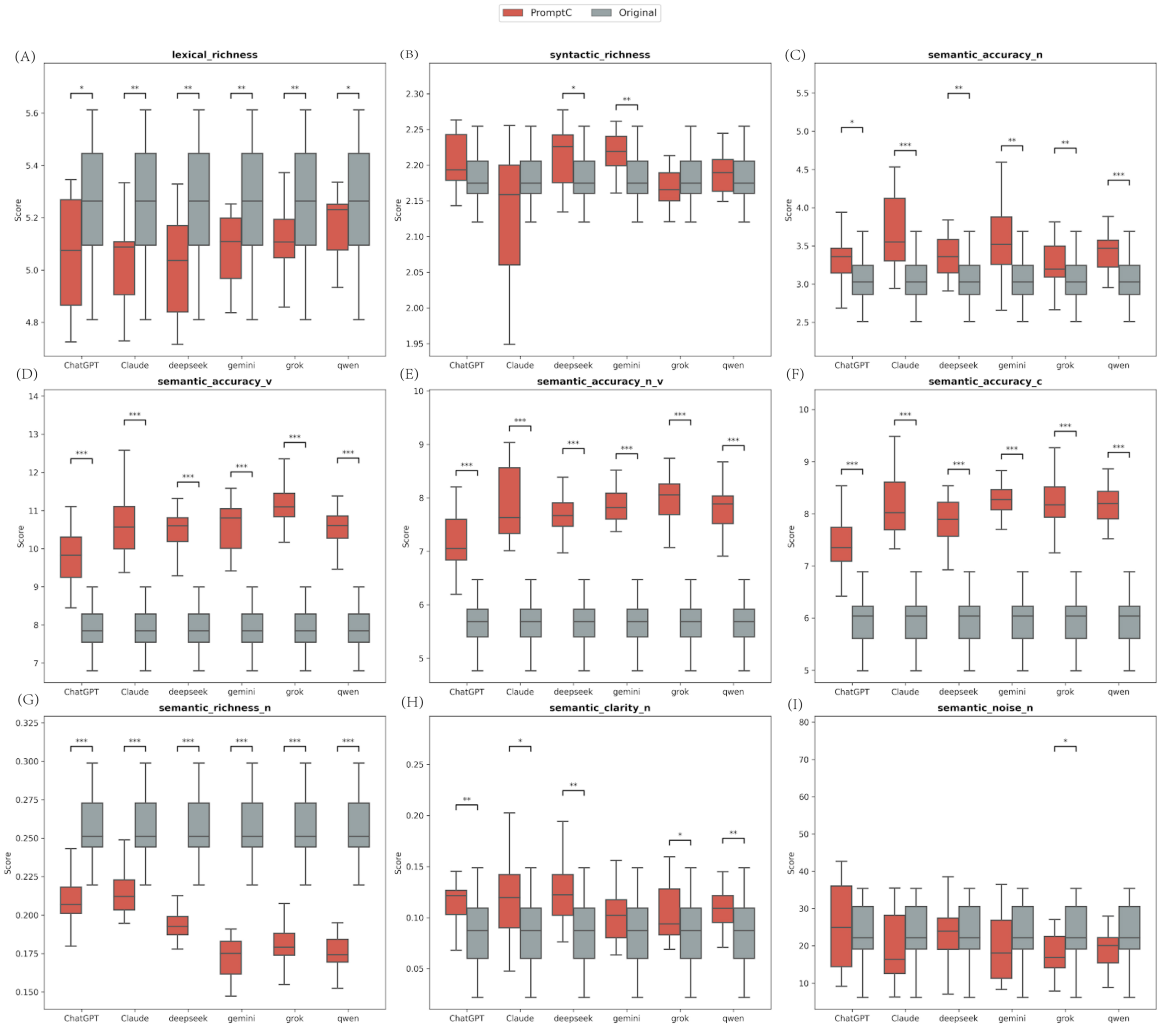
Comparison of linguistic features between AI-rewritten and original texts. The nine panels represent the nine linguistic feature indicators measured by the Alpha Readability Chinese (ARC) tool: **(A)** Lexical richness, measuring vocabulary diversity; **(B)** Syntactic richness, measuring sentence structural complexity; **(C)** Semantic accuracy for nouns (Semantic accuracy-N); **(D)** Semantic accuracy for verbs (Semantic accuracy-V); **(E)** Combined noun and verb semantic accuracy (Semantic accuracy-N_V); **(F)** Overall comprehensive semantic accuracy (Semantic accuracy-C); **(G)** Semantic richness for nouns (Semantic richness-N); **(H)** Semantic clarity for nouns (Semantic clarity-N); **(I)** Semantic noise for nouns (Semantic noise-N), measuring redundant or distracting information (lower is better). ***Indicates *p* < 0.001, **Indicates *p* < 0.01, and *Indicates *p* < 0.05.

We compared the 20 original articles with the texts rewritten by the AI models (Prompt C). We used the Wilcoxon Signed-Rank Test. The results showed clear differences in the structure of the text (Figure 10; Supplementary Table S25).

First, all six models scored much higher than the original texts in semantic accuracy (*p* < 0.001). This included accuracy for verbs, nouns, and the overall score. For example, the original texts had a median accuracy score of 6.04. The AI models ranged from 7.35 to 8.27. Also, most models produced clearer text (Semantic Clarity) than the originals (*p* < 0.05). The only exception was Gemini.

However, this improvement had a downside. The information density dropped. All models used a smaller variety of words (Lexical Richness) and fewer distinct nouns (Semantic Richness) compared to the original texts (*p* < 0.05).

We also looked at semantic noise. Only Grok significantly lowered the noise level (Md = 16.88) compared to the original texts (Md = 22.16, *p* = 0.044). The other models did not show a significant drop in noise.

In summary, Prompt C effectively refined the original text. It significantly improved surrogate metrics of accuracy and clarity, though this was accompanied by reduced vocabulary diversity.

## 5 Discussion

### 5.1 Key findings

This study systematically evaluated the ability of six major Large Language Models (LLMs) to create and improve health education materials for Neonatal Home Oxygen Therapy (NHOT). We found that while all models maintained high medical accuracy, they differed significantly in quality, understandability, and actionability. LLMs show great potential to make patient education materials easier to read while keeping them accurate and understandable (Will et al., 2025). This study confirms this finding and is the first to systematically evaluate this in the Chinese medical context for neonatal home oxygen therapy.

It is worth noting that the prompt strategy designed to “simplify” content (Prompt B) lowered the professional quality score significantly. Surprisingly, it did not improve understandability or actionability as expected. This matches the findings of Ellison et al. (2025), suggesting that prompt engineering is key to improving readability, but different strategies produce very different results. This shows that simply asking the model to “use simple language” is not enough to optimize education materials. In fact, it might sacrifice the completeness and depth of the content.

### 5.2 AI optimization of existing materials

One of the most important findings of this study is that all six models significantly improved the understandability of the original WeChat science articles during the rewriting task (*p* < 0.01). This result aligns with several other studies. Research shows that using ChatGPT with simple prompts to rewrite patient manuals significantly improves readability scores (Swisher et al., 2024). In the Chinese context, this is very practical. For example, education materials for Lupus patients in China are often hard to read and need simpler words and shorter sentences (Wang et al., 2020).

The use of neonatal home oxygen therapy is growing fast in China. Recently, it has been used more for infants with bronchopulmonary dysplasia. However, in the past, acceptance was low due to equipment costs, difficult care, or parental anxiety (Lin et al., 2022). In this context, high-quality, easy-to-understand materials are essential to boost parent confidence and ensure safety. This study proves that AI rewriting can effectively improve the readability of existing materials, offering a practical solution for this clinical need.

### 5.3 Complexity and limitations of prompt engineering

This study reveals the complexity of prompt engineering for medical education materials. The shift from Prompt A to Prompt B created a significant trade-off: while semantic accuracy improved and “noise” decreased, the quality score (DISCERN) dropped sharply, as did vocabulary richness. In research on simplifying cardiovascular disease information, the importance of optimizing prompts and using expert judgment was highlighted. Future guidelines should cover best practices for prompt engineering and standard evaluation of model outputs (Mishra et al., 2024).

Prompt engineering faces many challenges. Prompts based on the same framework can yield different answers with just small word changes, and different models react differently (Wang et al., 2024). Our findings support this: even a carefully designed “simplification” prompt did not achieve the desired results in all areas. This suggests that when using LLMs in health education, we need to develop more detailed strategies targeted at specific groups, rather than simply trying to “simplify” the language.

Research shows that prompt design is the most common method in medical AI studies (Zaghir et al., 2024). However, this study shows that design must balance language simplicity with professional content, completeness, and usability. Future research should explore multi-step strategies, such as chain-of-thought prompting or Retrieval-Augmented Generation.

### 5.4 Model differences and clinical implications

The six models showed significant performance differences in the baseline condition, which has important clinical implications. Qwen performed best in DISCERN quality scores, while Claude achieved full scores in actionability. This indicates that different models have different strengths. In a study on pediatric orthopedic materials, ChatGPT-4.0 and Google Gemini differed in their effectiveness at improving readability in English and Spanish (Nian et al., 2025).

In the rewriting task, the performance of the models tended to become similar (“assimilation”). This is worth discussing. When given a specific text to rewrite, the models produced more uniform output. This may mean that the rewriting task provides clearer context and rules for the models. This suggests that in real-world applications, using LLMs to optimize existing materials may be more reliable and consistent than generating materials from scratch.

### 5.5 Insights from linguistic feature analysis

We used the Alpha Readability Chinese (ARC) tool to analyze the linguistic features of the text. This revealed a consistent pattern when moving from Prompt A to Prompt B. All models showed the same trend: verb accuracy and overall semantic accuracy improved significantly, and semantic noise decreased. However, both vocabulary richness and semantic richness dropped.

This trade-off between “accuracy and richness” shows how the models work during optimization. To improve accuracy and make the text easier to read, the models tend to use a smaller range of words, favoring common and high-frequency expressions. However, simplifying too much can lead to a loss of depth. This is likely the reason why the DISCERN quality scores dropped for the Prompt B group.

Crucially, we compared the original texts with the AI-rewritten texts (Prompt C) to see how AI changes the deep structure of the text. The results showed that all six models performed significantly better than the original human-written texts in terms of semantic accuracy. This included accuracy for verbs, nouns, and the overall score. The median accuracy score for the original texts was only 6.04, while the AI models improved this to between 7.35 and 8.27. This finding proves that LLMs do not just simplify language; they systematically improve the precision of medical terms. This is vital for reducing patient misunderstanding and making health education materials more effective.

In addition, most models produced text with significantly better Semantic Clarity than the originals. The only exception was Gemini. This indicates that AI rewriting can effectively remove extra words and optimize sentence structure to make the core message stand out. However, this optimization comes at a cost. The Lexical Richness and Semantic Richness (Nouns) of all models were significantly lower than the original text. This drop in information density reflects a natural conflict in simplification strategies: making text easier to read often means sacrificing the variety of expression and the richness of detail.

It is worth noting that regarding semantic noise, only Grok significantly reduced the noise level (Md = 16.88 vs. Original Md = 22.16, *p* = 0.044). The other models did not show a significant difference. This suggests that models differ in their ability to “purify” information. Grok was able to remove distracting information while keeping the content clear. This ability is especially important when creating materials for people with low health literacy. Too much redundant information can increase cognitive load and make it harder to understand and remember key points.

These findings are especially important in the context of Chinese medical education. The Chinese version of the Patient Education Materials Assessment Tool (C-PEMAT) has been proven effective and reliable (Shan et al., 2023a). By combining objective semantic analysis with subjective tools like C-PEMAT, we can get a complete picture of the content generated by LLMs. The innovation of this study is that it combines objective and subjective evaluations to reveal the internal tension between “increased accuracy” and “decreased richness.” This provides important data for optimizing prompt strategies. Future prompt designs should clearly balance accuracy, clarity, and variety to avoid losing information through over-simplification.

Overall, Prompt C effectively streamlined the original text by improving accuracy and clarity while reducing vocabulary diversity. In specialized health scenarios like neonatal home oxygen therapy, this structural refinement is likely beneficial. The target audience (especially grandparent caregivers) needs accurate, clear, and focused information, not complex or varied expressions that might cause confusion. However, for scenarios that require comprehensive information, this type of simplification should be used with caution.

### 5.6 Medical accuracy and hallucination risk

An important finding is that all models maintained high medical accuracy across all three prompt strategies, with no significant differences between them. This contrasts with some other studies. In clinical note generation, a hallucination rate of 1.47% was observed (Asgari et al., 2025). In medical summary generation, hallucinations were found in almost all abstracts (Adams, 2024).

The low error rate in this study might be due to several factors. First, knowledge about neonatal home oxygen therapy is relatively mature and standard. Second, we evaluated educational materials for patients, not clinical advice for doctors, which lowers the risk of severe errors. Third, the 5-point Likert scale used by reviewers might not be as sensitive as specific hallucination detection tools.

However, large language models basically do not care about real-world facts. Their statistical predictions should not be treated the same as human reasoning based on evidence. This is especially important in medicine (Bélisle-Pipon, 2024). Research shows that when false details are included in a prompt, leading models often repeat the errors (Omar et al., 2025). Therefore, even though the models performed well here, strict review by medical professionals is still required in practice.

Beyond factual accuracy, the use of AI in health education raises significant ethical concerns regarding misinformation and the potential for harmful hallucinations. Therefore, strict ethical safeguards must be implemented. AI-generated patient materials must never bypass human oversight; a mandatory review process by qualified clinical professionals is essential before any material is distributed to patients. Furthermore, clear disclaimers stating that the content is AI-assisted and does not replace professional medical advice should be uniformly applied.

### 5.7 Special challenges for Chinese NHOT education

Neonatal home oxygen therapy faces unique challenges in China. A multi-center study showed that its use varies greatly across the country and is negatively related to local economic levels. There are no unified evidence-based guidelines (Jiang et al., 2023). Education teams typically need 2 weeks to teach parents skills like feeding and monitoring. If parents are confident, the team provides extra training on oxygen use (Lin et al., 2022).

In this context, high-quality, easy-to-understand materials are critical. Caregivers in China often include grandparents with lower education levels who may find medical terms confusing. Reviews show that most patient materials are harder to read than the recommended level (Okuhara et al., 2025). This study demonstrates that AI technology can lower the assessed reading difficulty of existing materials, providing a potential new tool to help address this long-standing problem.

It is crucial to interpret these findings with caution. Our conclusions are based entirely on surrogate metrics, including DISCERN, C-PEMAT, and the ARC tool. Because actual patient comprehension, subsequent behavioral changes, and clinical safety outcomes were not evaluated in the target caregiver population, our results must be interpreted at a descriptive level rather than as causal evidence. The AI-optimized texts generated in this study represent a promising strategy for content adaptation, but they should not yet be viewed as a ready-to-implement clinical solution without further real-world validation.

### 5.8 Limitations

This study has several limitations. The sample was modest (20 texts per model per strategy), which constrains statistical power and amplifies the influence of idiosyncratic texts. The rating process also depended on two reviewers; strong inter-rater reliability reduces concern, but it does not eliminate subjective drift in judgments that inevitably involve interpretation.

A more consequential design issue concerns comparability across prompt strategies. The source material was not held constant: Prompts A and B elicited *de novo* drafts, whereas Prompt C rewrote clinician-authored articles. Differences observed in readability, clarity, or linguistic features may therefore index baseline variation in the inputs—topic framing, authorial style, and intrinsic complexity—rather than the causal effect of the prompting strategy itself.

The evaluation framework also omitted an explicit measure of content completeness. Although we scored semantic accuracy and clarity, neither metric guarantee that key instructions, contraindications, or qualifying details survived simplification. As a result, an output could score well while quietly dropping clinically relevant elements. Finally, we did not solicit feedback from the intended users (caregivers). Without end-user testing, the relationship between our computed semantic measures and real comprehension, decision-making, or behavior change remains inferential rather than demonstrated.

### 5.9 Recommendations and future directions

These findings support three clinically practical moves. First, treat LLMs as revisers, not originators: they perform more reliably when polishing materials that have already been medically vetted than when drafting patient education from a blank prompt. Second, keep clinicians in the loop. They should shape the prompt, specify what must not be omitted, and review outputs before release. Third, handle “simplification” as a constrained operation rather than a stylistic preference. If the only instruction is “use simple language,” the model often buys readability by deleting content; prompts need explicit coverage requirements and guardrails.

The study’s scope also limits what can be claimed. Because we tested a single, narrow topic (NHOT), it remains unclear how well these strategies transfer to other high-complexity domains such as adult oncology or general pediatrics, or to multilingual workflows. Follow-on work should therefore stress-test the same prompting approaches across conditions and languages, and it should measure completeness directly—through content-overlap scoring, expert coverage annotation, or comparable methods—rather than assuming it tracks with readability. The endpoint should be clinical rather than purely textual: prospective studies are needed to determine whether these revisions measurably improve patient comprehension and health literacy in real care settings.

## 6 Conclusion

This study systematically evaluated six major large language models for generating and optimizing neonatal home oxygen therapy materials. It confirmed the significant potential of AI to improve the readability of existing materials. The study revealed the complexity of prompt engineering, showing that simple “simplification” strategies have trade-offs. The differences between models and the “assimilation” in rewriting tasks provide important references for real-world use. Although all models maintained high accuracy, strict human-AI collaboration is still needed. In the context of the challenges facing Chinese neonatal care, AI offers new possibilities to improve health literacy, but its safety and effectiveness need further verification.

## Data Availability

The raw data supporting the conclusions of this article will be made available by the authors, without undue reservation.
