# Correction: Evaluation of large language models in generating and optimizing educational materials for neonatal home oxygen therapy

**DOI:** 10.3389/frai.2026.1842850

**Published:** 2026-04-13

**Authors:** Zhendong Liu, Xiaoping Yang, Yu Zhang, Yujing Xu, Yue Xiang, Hongyan Wang

**Affiliations:** 1 School of Nursing, Gansu University of Chinese Medicine, Lanzhou, China; 2 Department of Neonatology, Gansu Provincial Maternal and Child Health Hospital, Lanzhou, China

**Keywords:** bronchopulmonary dysplasia, health education materials, large language models, neonatal home oxygen therapy, patient education, prompt engineering, readability

The reference for Dehkordi et al. (2025) was erroneously written as Dehkordi M. K. H., Lu J., Perl Y., Deek F. P. editors (2025). Enhancing patient comprehension of discharge notes with a retrieval-augmented LLM approach. IEEE International Conference on Bioinformatics and Biomedicine (BIBM), Piscataway, NJ: IEEE. It should be Dehkordi, M. K. H., Lu, J., Perl, Y., and Deek, F. P. (2025). “Enhancing patient comprehension of discharge notes with a retrieval-augmented LLM approach,” in *2025 IEEE International Conference on Bioinformatics and Biomedicine (BIBM)* (Wuhan: IEEE), 6966–6973, doi: 10.1109/BIBM66473.2025.11356919.

The reference for Dehkordi et al. (2024) was erroneously written as Dehkordi, M. K. H., Zhou, S., Perl, Y., Deek, F. P., Einstein, A. J., Elhanan, G., et al. editors (2024). Enhancing patient Comprehension: An effective sequential prompting approach to simplifying EHRs using LLMs. *2024 IEEE International Conference on Bioinformatics and Biomedicine (BIBM)*, Piscataway, NJ: IEEE. It should be Dehkordi, M. K. H., Zhou, S., Perl, Y., Deek, F. P., Einstein, A. J., Elhanan, G., et al. (2024). “Enhancing patient comprehension: an effective sequential prompting approach to simplifying EHRs using LLMs,” in *2024 IEEE International Conference on Bioinformatics and Biomedicine (BIBM)* (Lisbon: IEEE), 6370–6377, doi: 10.1109/BIBM62325.2024.10822313.

The original version of this article has been updated.

