# Supplementary material for: Evaluation of large language models in generating and optimizing educational materials for neonatal home oxygen therapy
Source: Front Artif Intell. 2026 Mar 27;9:1770564. doi: 10.3389/frai.2026.1770564 (PMC13066276; doi:10.3389/frai.2026.1770564)
Supplement: Supplementary file 1 [file Table_1.DOCX]

Table S1. Overall Performance Differences Among Six Models Under Baseline Condition (Prompt A).

| Indicator | H-statistic | p-value | Significant |
| --- | --- | --- | --- |
| Likert | 2.05256 | 0.841825 | NO |
| DISCERN | 44.032 | 2.28E-08 | YES |
| understandability | 23.4177 | 2.81E-04 | YES |
| actionability | 25.454 | 1.14E-04 | YES |

Table S2. Pairwise Comparisons of Medical Accuracy (Likert) Under Baseline Condition (Prompt A).

| Model 1 | Model 2 | U-statistic | p-value | p-adj (Bonferroni) | Significant |
| --- | --- | --- | --- | --- | --- |
| ChatGPT | Claude | 190 | 0.342 | 1 | NO |
| ChatGPT | Deepseek | 200 | 1 | 1 | NO |
| ChatGPT | Gemini | 190 | 0.342 | 1 | NO |
| ChatGPT | Grok | 190 | 0.342 | 1 | NO |
| ChatGPT | Qwen | 190 | 0.342 | 1 | NO |
| Claude | Deepseek | 210 | 0.342 | 1 | NO |
| Claude | Gemini | 200 | 1 | 1 | NO |
| Claude | Grok | 199.5 | 1 | 1 | NO |
| Claude | Qwen | 200 | 1 | 1 | NO |
| Deepseek | Gemini | 190 | 0.342 | 1 | NO |
| Deepseek | Grok | 190 | 0.342 | 1 | NO |
| Deepseek | Qwen | 190 | 0.342 | 1 | NO |
| Gemini | Grok | 199.5 | 1 | 1 | NO |
| Gemini | Qwen | 200 | 1 | 1 | NO |
| Grok | Qwen | 200.5 | 1 | 1 | NO |

Table S3. Pairwise Comparisons of Content Quality (DISCERN) Under Baseline Condition (Prompt A).

| Model 1 | Model 2 | U-statistic | p-value | p-adj (Bonferroni) | Significant |
| --- | --- | --- | --- | --- | --- |
| ChatGPT | CLAUDE | 130.5 | 0.0602 | 0.903 | NO |
| ChatGPT | Deepseek | 280.5 | 0.0297 | 0.445 | NO |
| ChatGPT | Gemini | 227 | 0.4708 | 1 | NO |
| ChatGPT | Grok | 159.5 | 0.2759 | 1 | NO |
| ChatGPT | Qwen | 52.5 | 6.64E-05 | 0.001 | YES |
| Claude | Deepseek | 340 | 0.0001 | 0.002 | YES |
| Claude | Gemini | 304.5 | 0.0045 | 0.067 | NO |
| Claude | Grok | 231 | 0.4019 | 1 | NO |
| Claude | Qwen | 88 | 0.0023 | 0.035 | YES |
| Deepseek | Gemini | 130.5 | 0.0607 | 0.91 | NO |
| Deepseek | Grok | 81 | 0.0013 | 0.019 | YES |
| Deepseek | Qwen | 20 | 1.13E-06 | 1.70E-05 | YES |
| Gemini | Grok | 129 | 0.0538 | 0.808 | NO |
| Gemini | Qwen | 36 | 8.74E-06 | 0.0001 | YES |
| Grok | Qwen | 71 | 0.0004 | 0.007 | YES |

Table S4. Pairwise Comparisons of Understandability Under Baseline Condition (Prompt A).

| Model 1 | Model 2 | U-statistic | p-value | p-adj (Bonferroni) | Significant |
| --- | --- | --- | --- | --- | --- |
| ChatGPT | Claude | 156 | 0.1699 | 1 | NO |
| ChatGPT | Deepseek | 265 | 0.0665 | 0.997 | NO |
| ChatGPT | Gemini | 168 | 0.3306 | 1 | NO |
| ChatGPT | Grok | 131 | 0.0243 | 0.365 | NO |
| ChatGPT | Qwen | 127 | 0.0167 | 0.251 | NO |
| Claude | Deepseek | 292.5 | 0.0068 | 0.103 | NO |
| Claude | Gemini | 215 | 0.6255 | 1 | NO |
| Claude | Grok | 178.5 | 0.4153 | 1 | NO |
| Claude | Qwen | 177 | 0.3821 | 1 | NO |
| Deepseek | Gemini | 109.5 | 0.0092 | 0.138 | NO |
| Deepseek | Grok | 80 | 0.0003 | 0.005 | YES |
| Deepseek | Qwen | 78 | 0.0002 | 0.004 | YES |
| Gemini | Grok | 161.5 | 0.1735 | 1 | NO |
| Gemini | Qwen | 158.5 | 0.1408 | 1 | NO |
| Grok | Qwen | 198.5 | 0.9652 | 1 | NO |

Table S5. Pairwise Comparisons of Actionability Under Baseline Condition (Prompt A).

| Model 1 | Model 2 | U-statistic | p-value | p-adj (Bonferroni) | Significant |
| --- | --- | --- | --- | --- | --- |
| ChatGPT | Claude | 70 | 3.00E-05 | 0.0004 | YES |
| ChatGPT | Deepseek | 97 | 0.0013 | 0.02 | YES |
| ChatGPT | Gemini | 140 | 0.0619 | 0.928 | NO |
| ChatGPT | Grok | 170 | 0.3414 | 1 | NO |
| ChatGPT | Qwen | 110 | 0.0051 | 0.077 | NO |
| Claude | Deepseek | 220.5 | 0.2985 | 1 | NO |
| Claude | Gemini | 270 | 0.0094 | 0.14 | NO |
| Claude | Grok | 300 | 0.0007 | 0.01 | YES |
| Claude | Qwen | 240 | 0.0842 | 1 | NO |
| Deepseek | Gemini | 246 | 0.1132 | 1 | NO |
| Deepseek | Grok | 274.5 | 0.0161 | 0.241 | NO |
| Deepseek | Qwen | 217.5 | 0.5081 | 1 | NO |
| Gemini | Grok | 230 | 0.3564 | 1 | NO |
| Gemini | Qwen | 170 | 0.3254 | 1 | NO |
| Grok | Qwen | 140 | 0.0579 | 0.869 | NO |

Table S6. Overall Performance Differences Among Six Models Under Simplification Strategy (Prompt B).

| Indicator | H-statistic | p-value | Significant |
| --- | --- | --- | --- |
| Likert | 4.11 | 0.534 | NO |
| DISCERN | 42.26 | 5.21E-08 | YES |
| understandability | 8.21 | 0.145 | NO |
| actionability | 37.23 | 5.39E-07 | YES |

Table S7. Pairwise Comparisons of Medical Accuracy (Likert) Under Simplification Strategy (Prompt B).

| Model 1 | Model 2 | U-statistic | p-value | p-adj (Bonferroni) | Significant |
| --- | --- | --- | --- | --- | --- |
| ChatGPT | Claude | 190 | 0.697 | 1 | NO |
| ChatGPT | Deepseek | 200 | 1 | 1 | NO |
| ChatGPT | Gemini | 230 | 0.081 | 1 | NO |
| ChatGPT | Grok | 207.5 | 0.742 | 1 | NO |
| ChatGPT | Qwen | 198 | 0.948 | 1 | NO |
| Claude | Deepseek | 210 | 0.697 | 1 | NO |
| Claude | Gemini | 240 | 0.04 | 0.6 | NO |
| Claude | Grok | 217.5 | 0.459 | 1 | NO |
| Claude | Qwen | 208 | 0.759 | 1 | NO |
| Deepseek | Gemini | 230 | 0.081 | 1 | NO |
| Deepseek | Grok | 207.5 | 0.742 | 1 | NO |
| Deepseek | Qwen | 198 | 0.948 | 1 | NO |
| Gemini | Grok | 180 | 0.163 | 1 | NO |
| Gemini | Qwen | 170 | 0.081 | 1 | NO |
| Grok | Qwen | 191 | 0.689 | 1 | NO |

Table S8. Pairwise Comparisons of Content Quality (DISCERN) Under Simplification Strategy (Prompt B).

| Model 1 | Model 2 | U-statistic | p-value | p-adj (Bonferroni) | Significant |
| --- | --- | --- | --- | --- | --- |
| ChatGPT | Claude | 49 | 4.43E-05 | 0.0007 | YES |
| ChatGPT | Deepseek | 151.5 | 0.191 | 1 | NO |
| ChatGPT | Gemini | 170.5 | 0.431 | 1 | NO |
| ChatGPT | Grok | 40 | 1.48E-05 | 0.0002 | YES |
| ChatGPT | Qwen | 205.5 | 0.892 | 1 | NO |
| Claude | Deepseek | 334.5 | 0.0003 | 0.004 | YES |
| Claude | Gemini | 328 | 0.0005 | 0.0077 | YES |
| Claude | Grok | 165 | 0.347 | 1 | NO |
| Claude | Qwen | 338 | 0.0002 | 0.0028 | YES |
| Deepseek | Gemini | 217.5 | 0.644 | 1 | NO |
| Deepseek | Grok | 52.5 | 6.27E-05 | 0.0009 | YES |
| Deepseek | Qwen | 243.5 | 0.242 | 1 | NO |
| Gemini | Grok | 65 | 0.0003 | 0.0039 | YES |
| Gemini | Qwen | 232.5 | 0.385 | 1 | NO |
| Grok | Qwen | 347.5 | 6.60E-05 | 0.001 | YES |

Table S9. Pairwise Comparisons of Understandability Under Simplification Strategy (Prompt B).

| Model 1 | Model 2 | U-statistic | p-value | p-adj (Bonferroni) | Significant |
| --- | --- | --- | --- | --- | --- |
| ChatGPT | Claude | 183 | 0.606 | 1 | NO |
| ChatGPT | Deepseek | 212.5 | 0.72 | 1 | NO |
| ChatGPT | Gemini | 169 | 0.319 | 1 | NO |
| ChatGPT | Grok | 156 | 0.133 | 1 | NO |
| ChatGPT | Qwen | 167.5 | 0.253 | 1 | NO |
| Claude | Deepseek | 229 | 0.39 | 1 | NO |
| Claude | Gemini | 174 | 0.409 | 1 | NO |
| Claude | Grok | 156 | 0.142 | 1 | NO |
| Claude | Qwen | 162 | 0.196 | 1 | NO |
| Deepseek | Gemini | 144 | 0.085 | 1 | NO |
| Deepseek | Grok | 126 | 0.019 | 0.289 | NO |
| Deepseek | Qwen | 134 | 0.036 | 0.541 | NO |
| Gemini | Grok | 180 | 0.482 | 1 | NO |
| Gemini | Qwen | 179 | 0.447 | 1 | NO |
| Grok | Qwen | 196 | 0.886 | 1 | NO |

Table S10. Pairwise Comparisons of Actionability Under Simplification Strategy (Prompt B).

| Model 1 | Model 2 | U-statistic | p-value | p-adj (Bonferroni) | Significant |
| --- | --- | --- | --- | --- | --- |
| ChatGPT | Claude | 20.5 | 3.31E-08 | 5.00E-07 | YES |
| ChatGPT | Deepseek | 73 | 6.33E-05 | 0.0009 | YES |
| ChatGPT | Gemini | 125.5 | 0.01 | 0.15 | NO |
| ChatGPT | Grok | 157 | 0.098 | 1 | NO |
| ChatGPT | Qwen | 115 | 0.004 | 0.063 | NO |
| Claude | Deepseek | 250 | 0.042 | 0.629 | NO |
| Claude | Gemini | 300 | 0.0007 | 0.0105 | YES |
| Claude | Grok | 330 | 3.00E-05 | 0.0004 | YES |
| Claude | Qwen | 290 | 0.0018 | 0.0263 | YES |
| Deepseek | Gemini | 250 | 0.118 | 1 | NO |
| Deepseek | Grok | 280 | 0.013 | 0.196 | NO |
| Deepseek | Qwen | 240 | 0.208 | 1 | NO |
| Gemini | Grok | 230 | 0.341 | 1 | NO |
| Gemini | Qwen | 190 | 0.766 | 1 | NO |
| Grok | Qwen | 160 | 0.208 | 1 | NO |

Table S11. Overall Performance Differences Among Six Models Under Rewriting Task (Prompt C).

| Indicator | H-statistic | p-value | Significant |
| --- | --- | --- | --- |
| Likert | 4.19098 | 0.522261 | NO |
| DISCERN | 7.69637 | 0.173782 | NO |
| understandability | 22.7947 | 0.000369 | YES |
| actionability | 7.69344 | 0.17396 | NO |

Table S12. Pairwise Comparisons of Medical Accuracy (Likert) Under Rewriting Task (Prompt C).

| Model 1 | Model 2 | U-statistic | p-value | p-adj (Bonferroni) | Significant |
| --- | --- | --- | --- | --- | --- |
| ChatGPT | Claude | 182 | 0.591 | 1 | NO |
| ChatGPT | Deepseek | 200.5 | 1 | 1 | NO |
| ChatGPT | Gemini | 226.5 | 0.384 | 1 | NO |
| ChatGPT | Grok | 232 | 0.278 | 1 | NO |
| ChatGPT | Qwen | 206 | 0.861 | 1 | NO |
| Claude | Deepseek | 220 | 0.55 | 1 | NO |
| Claude | Gemini | 247.5 | 0.133 | 1 | NO |
| Claude | Grok | 252.5 | 0.09 | 1 | NO |
| Claude | Qwen | 227.5 | 0.407 | 1 | NO |
| Deepseek | Gemini | 229 | 0.34 | 1 | NO |
| Deepseek | Grok | 233 | 0.261 | 1 | NO |
| Deepseek | Qwen | 209 | 0.785 | 1 | NO |
| Gemini | Grok | 206 | 0.838 | 1 | NO |
| Gemini | Qwen | 175.5 | 0.421 | 1 | NO |
| Grok | Qwen | 172.5 | 0.351 | 1 | NO |

Table S13. Pairwise Comparisons of Content Quality (DISCERN) Under Rewriting Task (Prompt C).

| Model 1 | Model 2 | U-statistic | p-value | p-adj (Bonferroni) | Significant |
| --- | --- | --- | --- | --- | --- |
| ChatGPT | Claude | 208.5 | 0.828 | 1 | NO |
| ChatGPT | Deepseek | 184.5 | 0.684 | 1 | NO |
| ChatGPT | Gemini | 181 | 0.616 | 1 | NO |
| ChatGPT | Grok | 135 | 0.08 | 1 | NO |
| ChatGPT | Qwen | 136 | 0.085 | 1 | NO |
| Claude | Deepseek | 170.5 | 0.432 | 1 | NO |
| Claude | Gemini | 169.5 | 0.416 | 1 | NO |
| Claude | Grok | 134.5 | 0.078 | 1 | NO |
| Claude | Qwen | 124.5 | 0.042 | 0.631 | NO |
| Deepseek | Gemini | 197 | 0.946 | 1 | NO |
| Deepseek | Grok | 153.5 | 0.212 | 1 | NO |
| Deepseek | Qwen | 145.5 | 0.143 | 1 | NO |
| Gemini | Grok | 151 | 0.188 | 1 | NO |
| Gemini | Qwen | 147.5 | 0.158 | 1 | NO |
| Grok | Qwen | 205 | 0.903 | 1 | NO |

Table S14. Pairwise Comparisons of Understandability Under Rewriting Task (Prompt C).

| Model 1 | Model 2 | U-statistic | p-value | p-adj (Bonferroni) | Significant |
| --- | --- | --- | --- | --- | --- |
| ChatGPT | Claude | 149.5 | 0.164 | 1 | NO |
| ChatGPT | Deepseek | 208.5 | 0.825 | 1 | NO |
| ChatGPT | Gemini | 106.5 | 0.0086 | 0.129 | NO |
| ChatGPT | Grok | 91.5 | 0.0021 | 0.032 | YES |
| ChatGPT | Qwen | 94 | 0.0027 | 0.041 | YES |
| Claude | Deepseek | 259 | 0.105 | 1 | NO |
| Claude | Gemini | 152.5 | 0.174 | 1 | NO |
| Claude | Grok | 138 | 0.072 | 1 | NO |
| Claude | Qwen | 141.5 | 0.091 | 1 | NO |
| Deepseek | Gemini | 100 | 0.0051 | 0.077 | NO |
| Deepseek | Grok | 87 | 0.0014 | 0.022 | YES |
| Deepseek | Qwen | 89 | 0.0018 | 0.027 | YES |
| Gemini | Grok | 186 | 0.68 | 1 | NO |
| Gemini | Qwen | 189.5 | 0.761 | 1 | NO |
| Grok | Qwen | 203.5 | 0.926 | 1 | NO |

Table S15. Pairwise Comparisons of Actionability Under Rewriting Task (Prompt C).

| Model 1 | Model 2 | U-statistic | p-value | p-adj (Bonferroni) | Significant |
| --- | --- | --- | --- | --- | --- |
| ChatGPT | Claude | 167 | 0.277 | 1 | NO |
| ChatGPT | Deepseek | 192 | 0.759 | 1 | NO |
| ChatGPT | Gemini | 182 | 0.446 | 1 | NO |
| ChatGPT | Grok | 137 | 0.03 | 0.452 | NO |
| ChatGPT | Qwen | 155 | 0.124 | 1 | NO |
| Claude | Deepseek | 227 | 0.361 | 1 | NO |
| Claude | Gemini | 220 | 0.487 | 1 | NO |
| Claude | Grok | 170 | 0.352 | 1 | NO |
| Claude | Qwen | 186.5 | 0.683 | 1 | NO |
| Deepseek | Gemini | 191 | 0.689 | 1 | NO |
| Deepseek | Grok | 143.5 | 0.044 | 0.664 | NO |
| Deepseek | Qwen | 161.5 | 0.174 | 1 | NO |
| Gemini | Grok | 150 | 0.064 | 0.963 | NO |
| Gemini | Qwen | 169 | 0.256 | 1 | NO |
| Grok | Qwen | 216.5 | 0.603 | 1 | NO |

Table S16. Impact of Prompt Strategy on Model Performance Metrics (Prompt A vs. Prompt B).

| Model | Indicator | U-statistic | p-value | Significant |
| --- | --- | --- | --- | --- |
| ChatGPT | DISCERN | 380.5 | 1.05 × 10⁻⁶ | YES |
| ChatGPT | Likert | 170 | 0.081 | NO |
| ChatGPT | Understandability | 191 | 0.797 | NO |
| ChatGPT | Actionability | 243 | 0.098 | NO |
| Claude | DISCERN | 374.5 | 2.19 × 10⁻⁶ | YES |
| Claude | Likert | 169.5 | 0.157 | NO |
| Claude | Understandability | 225 | 0.422 | NO |
| Claude | Actionability | 200 | 1 | NO |
| Deepseek | DISCERN | 322 | 0.00096 | YES |
| Deepseek | Likert | 170 | 0.081 | NO |
| Deepseek | Understandability | 135.5 | 0.07 | NO |
| Deepseek | Actionability | 227 | 0.323 | NO |
| Gemini | DISCERN | 369 | 4.87 × 10⁻⁶ | YES |
| Gemini | Likert | 210 | 0.342 | NO |
| Gemini | Understandability | 187 | 0.679 | NO |
| Gemini | Actionability | 230 | 0.356 | NO |
| Grok | DISCERN | 321.5 | 0.00099 | YES |
| Grok | Likert | 190.5 | 0.594 | NO |
| Grok | Understandability | 208 | 0.758 | NO |
| Grok | Actionability | 230 | 0.341 | NO |
| Qwen | DISCERN | 400 | 6.54 × 10⁻⁸ | YES |
| Qwen | Likert | 179 | 0.287 | NO |
| Qwen | Understandability | 204.5 | 0.862 | NO |
| Qwen | Actionability | 250 | 0.11 | NO |

Table S17. Comparison of Content Quality and Suitability Between AI-Rewritten Texts and Original Articles (Prompt C vs. Original).

| Model | Indicator | Median Difference (Model − Original) | Statistic | p-value | Significant |
| --- | --- | --- | --- | --- | --- |
| ChatGPT | Likert | −1.0 | 17.5 | 0.2955 | NO |
| ChatGPT | DISCERN | 1 | 61.5 | 0.2907 | NO |
| ChatGPT | Understandability | 8.5 | 23 | 0.0062 | YES |
| ChatGPT | Actionability | 0 | 3 | 0.2207 | NO |
| Claude | Likert | −1.0 | 20 | 0.7647 | NO |
| Claude | DISCERN | −1.5 | 83.5 | 0.6415 | NO |
| Claude | Understandability | 13.5 | 4 | 0.0004 | YES |
| Claude | Actionability | 0 | 12 | 0.2095 | NO |
| Deepseek | Likert | −1.0 | 18 | 0.3233 | NO |
| Deepseek | DISCERN | 1.5 | 47 | 0.09 | NO |
| Deepseek | Understandability | 11.0 | 15 | 0.006 | YES |
| Deepseek | Actionability | 0 | 4 | 0.7127 | NO |
| Gemini | Likert | −1.0 | 14.5 | 0.0508 | NO |
| Gemini | DISCERN | 1 | 86 | 0.498 | NO |
| Gemini | Understandability | 21.5 | 1.5 | 0.0002 | YES |
| Gemini | Actionability | 0 | 0 | 0.1025 | NO |
| Grok | Likert | −1.0 | 20 | 0.0698 | NO |
| Grok | DISCERN | 3.5 | 40.5 | 0.028 | YES |
| Grok | Understandability | 21.5 | 2 | 0.0003 | YES |
| Grok | Actionability | 0.0 | 0 | 0.0067 | YES |
| Qwen | Likert | −1.0 | 37.5 | 0.184 | NO |
| Qwen | DISCERN | 3.0 | 34 | 0.0245 | YES |
| Qwen | Understandability | 21.5 | 1.5 | 0.0002 | YES |
| Qwen | Actionability | 0.0 | 1 | 0.0128 | YES |

Table S18. Overall Linguistic Feature Differences Among Six Models Under Baseline Condition (Prompt A).

| Indicator | H_statistic | p_value | Significant |
| --- | --- | --- | --- |
| lexical_richness | 70.1748 | 9.42×10⁻¹⁴ | Yes |
| syntactic_richness | 91.3693 | 3.46×10⁻¹⁸ | Yes |
| semantic_accuracy_n | 40.7753 | 1.04×10⁻⁷ | Yes |
| semantic_accuracy_v | 86.7321 | 3.26×10⁻¹⁷ | Yes |
| semantic_accuracy_n_v | 80.2561 | 7.42×10⁻¹⁶ | Yes |
| semantic_accuracy_c | 79.5852 | 1.02×10⁻¹⁵ | Yes |
| semantic_richness_n | 89.9818 | 6.78×10⁻¹⁸ | Yes |
| semantic_clarity_n | 32.716 | 4.29×10⁻⁶ | Yes |
| semantic_noise_n | 58.7512 | 2.20×10⁻¹¹ | Yes |

Table S19. Pairwise Comparisons of Linguistic Features Among Six Models Under Baseline Condition (Prompt A).

| Indicator | Model_A | Model_B | p_value_uncorrected | p_value_corrected | Significant |
| --- | --- | --- | --- | --- | --- |
| lexical_richness | ChatGPT | Claude | 0.000000342 | 0.00000512 | Yes |
| lexical_richness | ChatGPT | deepseek | 0.072 | 1 | No |
| lexical_richness | ChatGPT | gemini | 0.0005 | 0.0069 | Yes |
| lexical_richness | ChatGPT | grok | 0.0021 | 0.0321 | Yes |
| lexical_richness | ChatGPT | qwen | 0.4407 | 1 | No |
| lexical_richness | Claude | deepseek | 0.000000143 | 0.00000215 | Yes |
| lexical_richness | Claude | gemini | 0.00000667 | 0.0001 | Yes |
| lexical_richness | Claude | grok | 0.0000018 | 0.000027 | Yes |
| lexical_richness | Claude | qwen | 0.000000166 | 0.00000249 | Yes |
| lexical_richness | deepseek | gemini | 0.000026 | 0.0004 | Yes |
| lexical_richness | deepseek | grok | 0.0001 | 0.0017 | Yes |
| lexical_richness | deepseek | qwen | 0.0207 | 0.311 | No |
| lexical_richness | gemini | grok | 0.0962 | 1 | No |
| lexical_richness | gemini | qwen | 0.0000292 | 0.0004 | Yes |
| lexical_richness | grok | qwen | 0.0002 | 0.0037 | Yes |
| syntactic_richness | ChatGPT | Claude | 0.0000035 | 0.0000525 | Yes |
| syntactic_richness | ChatGPT | deepseek | 0.000000222 | 0.00000333 | Yes |
| syntactic_richness | ChatGPT | gemini | 0.00000517 | 0.0000775 | Yes |
| syntactic_richness | ChatGPT | grok | 0.1636 | 1 | No |
| syntactic_richness | ChatGPT | qwen | 0.0003 | 0.0051 | Yes |
| syntactic_richness | Claude | deepseek | 0.0012 | 0.0184 | Yes |
| syntactic_richness | Claude | gemini | 0.000000068 | 0.00000102 | Yes |
| syntactic_richness | Claude | grok | 0.000000394 | 0.00000591 | Yes |
| syntactic_richness | Claude | qwen | 0.000000068 | 0.00000102 | Yes |
| syntactic_richness | deepseek | gemini | 0.000000068 | 0.00000102 | Yes |
| syntactic_richness | deepseek | grok | 9.17E-08 | 0.00000138 | Yes |
| syntactic_richness | deepseek | qwen | 0.000000068 | 0.00000102 | Yes |
| syntactic_richness | gemini | grok | 0.000059 | 0.0009 | Yes |
| syntactic_richness | gemini | qwen | 0.0315 | 0.4728 | No |
| syntactic_richness | grok | qwen | 0.0123 | 0.1852 | No |
| semantic_accuracy_n | ChatGPT | Claude | 0.00000454 | 0.0000681 | Yes |
| semantic_accuracy_n | ChatGPT | deepseek | 0.0001 | 0.0016 | Yes |
| semantic_accuracy_n | ChatGPT | gemini | 0.2184 | 1 | No |
| semantic_accuracy_n | ChatGPT | grok | 0.2733 | 1 | No |
| semantic_accuracy_n | ChatGPT | qwen | 0.1136 | 1 | No |
| semantic_accuracy_n | Claude | deepseek | 0.6168 | 1 | No |
| semantic_accuracy_n | Claude | gemini | 0.0026 | 0.0384 | Yes |
| semantic_accuracy_n | Claude | grok | 0.0000741 | 0.0011 | Yes |
| semantic_accuracy_n | Claude | qwen | 0.0004 | 0.0056 | Yes |
| semantic_accuracy_n | deepseek | gemini | 0.0028 | 0.042 | Yes |
| semantic_accuracy_n | deepseek | grok | 0.0001 | 0.0022 | Yes |
| semantic_accuracy_n | deepseek | qwen | 0.002 | 0.0293 | Yes |
| semantic_accuracy_n | gemini | grok | 0.0639 | 0.9584 | No |
| semantic_accuracy_n | gemini | qwen | 0.715 | 1 | No |
| semantic_accuracy_n | grok | qwen | 0.0066 | 0.0984 | No |
| semantic_accuracy_v | ChatGPT | Claude | 0.2977 | 1 | No |
| semantic_accuracy_v | ChatGPT | deepseek | 0.0016 | 0.0244 | Yes |
| semantic_accuracy_v | ChatGPT | gemini | 0.000000601 | 0.00000902 | Yes |
| semantic_accuracy_v | ChatGPT | grok | 0.000000068 | 0.00000102 | Yes |
| semantic_accuracy_v | ChatGPT | qwen | 0.9246 | 1 | No |
| semantic_accuracy_v | Claude | deepseek | 0.0000661 | 0.001 | Yes |
| semantic_accuracy_v | Claude | gemini | 0.00000158 | 0.0000236 | Yes |
| semantic_accuracy_v | Claude | grok | 0.000000068 | 0.00000102 | Yes |
| semantic_accuracy_v | Claude | qwen | 0.1806 | 1 | No |
| semantic_accuracy_v | deepseek | gemini | 0.000000068 | 0.00000102 | Yes |
| semantic_accuracy_v | deepseek | grok | 0.000000068 | 0.00000102 | Yes |
| semantic_accuracy_v | deepseek | qwen | 0.0071 | 0.1067 | No |
| semantic_accuracy_v | gemini | grok | 0.00000206 | 0.0000309 | Yes |
| semantic_accuracy_v | gemini | qwen | 0.000000342 | 0.00000512 | Yes |
| semantic_accuracy_v | grok | qwen | 0.000000068 | 0.00000102 | Yes |
| semantic_accuracy_n_v | ChatGPT | Claude | 0.0023 | 0.0351 | Yes |
| semantic_accuracy_n_v | ChatGPT | deepseek | 0.2733 | 1 | No |
| semantic_accuracy_n_v | ChatGPT | gemini | 0.000000454 | 0.00000681 | Yes |
| semantic_accuracy_n_v | ChatGPT | grok | 9.17E-08 | 0.00000138 | Yes |
| semantic_accuracy_n_v | ChatGPT | qwen | 0.081 | 1 | No |
| semantic_accuracy_n_v | Claude | deepseek | 0.0000741 | 0.0011 | Yes |
| semantic_accuracy_n_v | Claude | gemini | 0.0006 | 0.0084 | Yes |
| semantic_accuracy_n_v | Claude | grok | 0.000000692 | 0.0000104 | Yes |
| semantic_accuracy_n_v | Claude | qwen | 0.1333 | 1 | No |
| semantic_accuracy_n_v | deepseek | gemini | 0.000000068 | 0.00000102 | Yes |
| semantic_accuracy_n_v | deepseek | grok | 0.000000068 | 0.00000102 | Yes |
| semantic_accuracy_n_v | deepseek | qwen | 0.0033 | 0.05 | No |
| semantic_accuracy_n_v | gemini | grok | 0.001 | 0.0152 | Yes |
| semantic_accuracy_n_v | gemini | qwen | 0.00000454 | 0.0000681 | Yes |
| semantic_accuracy_n_v | grok | qwen | 0.000000166 | 0.00000249 | Yes |
| semantic_accuracy_c | ChatGPT | Claude | 0.0026 | 0.0384 | Yes |
| semantic_accuracy_c | ChatGPT | deepseek | 0.675 | 1 | No |
| semantic_accuracy_c | ChatGPT | gemini | 0.000000079 | 0.00000118 | Yes |
| semantic_accuracy_c | ChatGPT | grok | 0.000000068 | 0.00000102 | Yes |
| semantic_accuracy_c | ChatGPT | qwen | 0.0909 | 1 | No |
| semantic_accuracy_c | Claude | deepseek | 0.0006 | 0.0093 | Yes |
| semantic_accuracy_c | Claude | gemini | 0.0000181 | 0.0003 | Yes |
| semantic_accuracy_c | Claude | grok | 0.000000795 | 0.0000119 | Yes |
| semantic_accuracy_c | Claude | qwen | 0.2977 | 1 | No |
| semantic_accuracy_c | deepseek | gemini | 0.000000068 | 0.00000102 | Yes |
| semantic_accuracy_c | deepseek | grok | 0.000000068 | 0.00000102 | Yes |
| semantic_accuracy_c | deepseek | qwen | 0.0315 | 0.4728 | No |
| semantic_accuracy_c | gemini | grok | 0.0167 | 0.25 | No |
| semantic_accuracy_c | gemini | qwen | 0.00000158 | 0.0000236 | Yes |
| semantic_accuracy_c | grok | qwen | 0.000000523 | 0.00000784 | Yes |
| semantic_richness_n | ChatGPT | Claude | 0.1199 | 1 | No |
| semantic_richness_n | ChatGPT | deepseek | 0.0000661 | 0.001 | Yes |
| semantic_richness_n | ChatGPT | gemini | 0.000000601 | 0.00000901 | Yes |
| semantic_richness_n | ChatGPT | grok | 0.000000913 | 0.0000137 | Yes |
| semantic_richness_n | ChatGPT | qwen | 0.00000454 | 0.0000681 | Yes |
| semantic_richness_n | Claude | deepseek | 0.0008 | 0.0114 | Yes |
| semantic_richness_n | Claude | gemini | 6.79E-08 | 0.00000102 | Yes |
| semantic_richness_n | Claude | grok | 0.000000106 | 0.0000016 | Yes |
| semantic_richness_n | Claude | qwen | 9.17E-08 | 0.00000138 | Yes |
| semantic_richness_n | deepseek | gemini | 7.89E-08 | 0.00000118 | Yes |
| semantic_richness_n | deepseek | grok | 9.17E-08 | 0.00000138 | Yes |
| semantic_richness_n | deepseek | qwen | 0.000000106 | 0.0000016 | Yes |
| semantic_richness_n | gemini | grok | 0.0679 | 1 | No |
| semantic_richness_n | gemini | qwen | 0.4735 | 1 | No |
| semantic_richness_n | grok | qwen | 0.0123 | 0.1852 | No |
| semantic_clarity_n | ChatGPT | Claude | 0.6949 | 1 | No |
| semantic_clarity_n | ChatGPT | deepseek | 0.0565 | 0.8477 | No |
| semantic_clarity_n | ChatGPT | gemini | 0.0468 | 0.7019 | No |
| semantic_clarity_n | ChatGPT | grok | 0.0000141 | 0.0002 | Yes |
| semantic_clarity_n | ChatGPT | qwen | 0.1478 | 1 | No |
| semantic_clarity_n | Claude | deepseek | 0.0764 | 1 | No |
| semantic_clarity_n | Claude | gemini | 0.1333 | 1 | No |
| semantic_clarity_n | Claude | grok | 0.00000206 | 0.0000309 | Yes |
| semantic_clarity_n | Claude | qwen | 0.4735 | 1 | No |
| semantic_clarity_n | deepseek | gemini | 0.6554 | 1 | No |
| semantic_clarity_n | deepseek | grok | 0.0098 | 0.1468 | No |
| semantic_clarity_n | deepseek | qwen | 0.2184 | 1 | No |
| semantic_clarity_n | gemini | grok | 0.0005 | 0.0069 | Yes |
| semantic_clarity_n | gemini | qwen | 0.4735 | 1 | No |
| semantic_clarity_n | grok | qwen | 0.0000181 | 0.0003 | Yes |
| semantic_noise_n | ChatGPT | Claude | 0.000023 | 0.0003 | Yes |
| semantic_noise_n | ChatGPT | deepseek | 0.2085 | 1 | No |
| semantic_noise_n | ChatGPT | gemini | 0.00000975 | 0.0001 | Yes |
| semantic_noise_n | ChatGPT | grok | 0.0207 | 0.311 | No |
| semantic_noise_n | ChatGPT | qwen | 0.9246 | 1 | No |
| semantic_noise_n | Claude | deepseek | 0.0000371 | 0.0006 | Yes |
| semantic_noise_n | Claude | gemini | 0.3793 | 1 | No |
| semantic_noise_n | Claude | grok | 0.0000012 | 0.000018 | Yes |
| semantic_noise_n | Claude | qwen | 0.0000141 | 0.0002 | Yes |
| semantic_noise_n | deepseek | gemini | 0.0000526 | 0.0008 | Yes |
| semantic_noise_n | deepseek | grok | 0.7353 | 1 | No |
| semantic_noise_n | deepseek | qwen | 0.1895 | 1 | No |
| semantic_noise_n | gemini | grok | 0.000000256 | 0.00000384 | Yes |
| semantic_noise_n | gemini | qwen | 0.00000399 | 0.0000598 | Yes |
| semantic_noise_n | grok | qwen | 0.009 | 0.1357 | No |

Table S20. Overall Linguistic Feature Differences Among Six Models Under Simplification Strategy (Prompt B).

| Indicator | H_statistic | p_value | Significant |
| --- | --- | --- | --- |
| lexical_richness | 89.58 | 8.22×10⁻¹⁸ | Yes |
| syntactic_richness | 58.09 | 3.01×10⁻¹¹ | Yes |
| semantic_accuracy_n | 26.65 | 6.69×10⁻⁵ | Yes |
| semantic_accuracy_v | 14.56 | 0.012 | Yes |
| semantic_accuracy_n_v | 33.39 | 3.15×10⁻⁶ | Yes |
| semantic_accuracy_c | 35.97 | 9.61×10⁻⁷ | Yes |
| semantic_richness_n | 61.48 | 6.00×10⁻¹² | Yes |
| semantic_clarity_n | 56.69 | 5.85×10⁻¹¹ | Yes |
| semantic_noise_n | 35.61 | 1.14×10⁻⁶ | Yes |

Table S21. Pairwise Comparisons of Linguistic Features Among Six Models Under Simplification Strategy (Prompt B).

| Indicator | Model_1 | Model_2 | p_value_uncorrected | p_value_bonferroni | Significant |
| --- | --- | --- | --- | --- | --- |
| lexical_richness | ChatGPT | Claude | 6.80E-08 | 1.02E-06 | Yes |
| lexical_richness | ChatGPT | deepseek | 1.29E-04 | 0.002 | Yes |
| lexical_richness | ChatGPT | gemini | 6.80E-08 | 1.02E-06 | Yes |
| lexical_richness | ChatGPT | grok | 6.80E-08 | 1.02E-06 | Yes |
| lexical_richness | ChatGPT | qwen | 0.86 | 1 | No |
| lexical_richness | Claude | deepseek | 5.23E-07 | 7.84E-06 | Yes |
| lexical_richness | Claude | gemini | 0.002 | 0.024 | Yes |
| lexical_richness | Claude | grok | 2.75E-04 | 0.004 | Yes |
| lexical_richness | Claude | qwen | 6.80E-08 | 1.02E-06 | Yes |
| lexical_richness | deepseek | gemini | 1.79E-04 | 0.003 | Yes |
| lexical_richness | deepseek | grok | 0.006 | 0.097 | No |
| lexical_richness | deepseek | qwen | 7.64E-05 | 0.001 | Yes |
| lexical_richness | gemini | grok | 0.38 | 1 | No |
| lexical_richness | gemini | qwen | 6.80E-08 | 1.02E-06 | Yes |
| lexical_richness | grok | qwen | 6.80E-08 | 1.02E-06 | Yes |
| syntactic_richness | ChatGPT | Claude | 0.002 | 0.024 | Yes |
| syntactic_richness | ChatGPT | deepseek | 1.22E-04 | 0.002 | Yes |
| syntactic_richness | ChatGPT | gemini | 6.80E-08 | 1.02E-06 | Yes |
| syntactic_richness | ChatGPT | grok | 1.93E-05 | 2.89E-04 | Yes |
| syntactic_richness | ChatGPT | qwen | 0.015 | 0.224 | No |
| syntactic_richness | Claude | deepseek | 0.198 | 1 | No |
| syntactic_richness | Claude | gemini | 3.23E-05 | 4.85E-04 | Yes |
| syntactic_richness | Claude | grok | 0.231 | 1 | No |
| syntactic_richness | Claude | qwen | 6.80E-08 | 1.02E-06 | Yes |
| syntactic_richness | deepseek | gemini | 6.80E-08 | 1.02E-06 | Yes |
| syntactic_richness | deepseek | grok | 0.817 | 1 | No |
| syntactic_richness | deepseek | qwen | 4.60E-04 | 0.007 | Yes |
| syntactic_richness | gemini | grok | 1.63E-06 | 2.44E-05 | Yes |
| syntactic_richness | gemini | qwen | 9.75E-06 | 1.46E-04 | Yes |
| syntactic_richness | grok | qwen | 1.88E-04 | 0.003 | Yes |
| semantic_accuracy_n | ChatGPT | Claude | 1.58E-06 | 2.36E-05 | Yes |
| semantic_accuracy_n | ChatGPT | deepseek | 0.007 | 0.101 | No |
| semantic_accuracy_n | ChatGPT | gemini | 0.015 | 0.224 | No |
| semantic_accuracy_n | ChatGPT | grok | 0.147 | 1 | No |
| semantic_accuracy_n | ChatGPT | qwen | 0.021 | 0.311 | No |
| semantic_accuracy_n | Claude | deepseek | 0.006 | 0.09 | No |
| semantic_accuracy_n | Claude | gemini | 0.002 | 0.035 | Yes |
| semantic_accuracy_n | Claude | grok | 0.003 | 0.05 | No |
| semantic_accuracy_n | Claude | qwen | 0.004 | 0.06 | No |
| semantic_accuracy_n | deepseek | gemini | 0.86 | 1 | No |
| semantic_accuracy_n | deepseek | grok | 0.298 | 1 | No |
| semantic_accuracy_n | deepseek | qwen | 0.776 | 1 | No |
| semantic_accuracy_n | gemini | grok | 0.285 | 1 | No |
| semantic_accuracy_n | gemini | qwen | 0.86 | 1 | No |
| semantic_accuracy_n | grok | qwen | 0.38 | 1 | No |
| semantic_accuracy_v | ChatGPT | Claude | 0.796 | 1 | No |
| semantic_accuracy_v | ChatGPT | deepseek | 0.338 | 1 | No |
| semantic_accuracy_v | ChatGPT | gemini | 0.011 | 0.17 | No |
| semantic_accuracy_v | ChatGPT | grok | 0.148 | 1 | No |
| semantic_accuracy_v | ChatGPT | qwen | 0.004 | 0.054 | No |
| semantic_accuracy_v | Claude | deepseek | 0.441 | 1 | No |
| semantic_accuracy_v | Claude | gemini | 0.02 | 0.295 | No |
| semantic_accuracy_v | Claude | grok | 0.22 | 1 | No |
| semantic_accuracy_v | Claude | qwen | 0.006 | 0.097 | No |
| semantic_accuracy_v | deepseek | gemini | 0.086 | 1 | No |
| semantic_accuracy_v | deepseek | grok | 0.735 | 1 | No |
| semantic_accuracy_v | deepseek | qwen | 0.039 | 0.584 | No |
| semantic_accuracy_v | gemini | grok | 0.148 | 1 | No |
| semantic_accuracy_v | gemini | qwen | 0.838 | 1 | No |
| semantic_accuracy_v | grok | qwen | 0.108 | 1 | No |
| semantic_accuracy_n_v | ChatGPT | Claude | 0.001 | 0.015 | Yes |
| semantic_accuracy_n_v | ChatGPT | deepseek | 0.039 | 0.584 | No |
| semantic_accuracy_n_v | ChatGPT | gemini | 0.003 | 0.038 | Yes |
| semantic_accuracy_n_v | ChatGPT | grok | 0.047 | 0.702 | No |
| semantic_accuracy_n_v | ChatGPT | qwen | 0.002 | 0.032 | Yes |
| semantic_accuracy_n_v | Claude | deepseek | 0.081 | 1 | No |
| semantic_accuracy_n_v | Claude | gemini | 0.86 | 1 | No |
| semantic_accuracy_n_v | Claude | grok | 0.047 | 0.702 | No |
| semantic_accuracy_n_v | Claude | qwen | 0.366 | 1 | No |
| semantic_accuracy_n_v | deepseek | gemini | 0.198 | 1 | No |
| semantic_accuracy_n_v | deepseek | grok | 0.715 | 1 | No |
| semantic_accuracy_n_v | deepseek | qwen | 0.457 | 1 | No |
| semantic_accuracy_n_v | gemini | grok | 0.091 | 1 | No |
| semantic_accuracy_n_v | gemini | qwen | 0.547 | 1 | No |
| semantic_accuracy_n_v | grok | qwen | 0.231 | 1 | No |
| semantic_accuracy_c | ChatGPT | Claude | 0.004 | 0.057 | No |
| semantic_accuracy_c | ChatGPT | deepseek | 0.037 | 0.548 | No |
| semantic_accuracy_c | ChatGPT | gemini | 0.004 | 0.06 | No |
| semantic_accuracy_c | ChatGPT | grok | 0.024 | 0.366 | No |
| semantic_accuracy_c | ChatGPT | qwen | 0.001 | 0.009 | Yes |
| semantic_accuracy_c | Claude | deepseek | 0.245 | 1 | No |
| semantic_accuracy_c | Claude | gemini | 1 | 1 | No |
| semantic_accuracy_c | Claude | grok | 0.425 | 1 | No |
| semantic_accuracy_c | Claude | qwen | 0.441 | 1 | No |
| semantic_accuracy_c | deepseek | gemini | 0.338 | 1 | No |
| semantic_accuracy_c | deepseek | grok | 0.735 | 1 | No |
| semantic_accuracy_c | deepseek | qwen | 0.064 | 0.958 | No |
| semantic_accuracy_c | gemini | grok | 0.394 | 1 | No |
| semantic_accuracy_c | gemini | qwen | 0.49 | 1 | No |
| semantic_accuracy_c | grok | qwen | 0.156 | 1 | No |
| semantic_richness_n | ChatGPT | Claude | 0.015 | 0.224 | No |
| semantic_richness_n | ChatGPT | deepseek | 5.23E-07 | 7.84E-06 | Yes |
| semantic_richness_n | ChatGPT | gemini | 3.53E-05 | 5.29E-04 | Yes |
| semantic_richness_n | ChatGPT | grok | 1.48E-04 | 0.002 | Yes |
| semantic_richness_n | ChatGPT | qwen | 0.338 | 1 | No |
| semantic_richness_n | Claude | deepseek | 0.001 | 0.012 | Yes |
| semantic_richness_n | Claude | gemini | 0.008 | 0.126 | No |
| semantic_richness_n | Claude | grok | 0.019 | 0.284 | No |
| semantic_richness_n | Claude | qwen | 0.003 | 0.048 | Yes |
| semantic_richness_n | deepseek | gemini | 0.231 | 1 | No |
| semantic_richness_n | deepseek | grok | 0.338 | 1 | No |
| semantic_richness_n | deepseek | qwen | 1.63E-06 | 2.44E-05 | Yes |
| semantic_richness_n | gemini | grok | 0.655 | 1 | No |
| semantic_richness_n | gemini | qwen | 2.07E-04 | 0.003 | Yes |
| semantic_richness_n | grok | qwen | 0.001 | 0.018 | Yes |
| semantic_clarity_n | ChatGPT | Claude | 0.014 | 0.206 | No |
| semantic_clarity_n | ChatGPT | deepseek | 0.057 | 0.848 | No |
| semantic_clarity_n | ChatGPT | gemini | 0.012 | 0.185 | No |
| semantic_clarity_n | ChatGPT | grok | 6.80E-08 | 1.02E-06 | Yes |
| semantic_clarity_n | ChatGPT | qwen | 0.002 | 0.035 | Yes |
| semantic_clarity_n | Claude | deepseek | 0.573 | 1 | No |
| semantic_clarity_n | Claude | gemini | 0.903 | 1 | No |
| semantic_clarity_n | Claude | grok | 1.63E-06 | 2.44E-05 | Yes |
| semantic_clarity_n | Claude | qwen | 0.49 | 1 | No |
| semantic_clarity_n | deepseek | gemini | 0.615 | 1 | No |
| semantic_clarity_n | deepseek | grok | 3.53E-05 | 5.29E-04 | Yes |
| semantic_clarity_n | deepseek | qwen | 0.231 | 1 | No |
| semantic_clarity_n | gemini | grok | 6.80E-08 | 1.02E-06 | Yes |
| semantic_clarity_n | gemini | qwen | 0.441 | 1 | No |
| semantic_clarity_n | grok | qwen | 7.90E-08 | 1.18E-06 | Yes |
| semantic_noise_n | ChatGPT | Claude | 0.114 | 1 | No |
| semantic_noise_n | ChatGPT | deepseek | 0.024 | 0.366 | No |
| semantic_noise_n | ChatGPT | gemini | 0.004 | 0.054 | No |
| semantic_noise_n | ChatGPT | grok | 0.001 | 0.008 | Yes |
| semantic_noise_n | ChatGPT | qwen | 0.441 | 1 | No |
| semantic_noise_n | Claude | deepseek | 0.507 | 1 | No |
| semantic_noise_n | Claude | gemini | 0.081 | 1 | No |
| semantic_noise_n | Claude | grok | 6.79E-05 | 0.001 | Yes |
| semantic_noise_n | Claude | qwen | 0.425 | 1 | No |
| semantic_noise_n | deepseek | gemini | 0.311 | 1 | No |
| semantic_noise_n | deepseek | grok | 0.002 | 0.035 | Yes |
| semantic_noise_n | deepseek | qwen | 0.127 | 1 | No |
| semantic_noise_n | gemini | grok | 4.30E-04 | 0.006 | Yes |
| semantic_noise_n | gemini | qwen | 0.024 | 0.366 | No |
| semantic_noise_n | grok | qwen | 0.006 | 0.09 | No |

Table S22. Overall Linguistic Feature Differences Among Six Models Under Rewriting Task (Prompt C).

| Indicator | H_statistic | p_value | Significant |
| --- | --- | --- | --- |
| lexical_richness | 12.91 | 0.024 | Yes |
| syntactic_richness | 28.77 | 2.57×10⁻⁵ | Yes |
| semantic_accuracy_n | 7.61 | 0.179 | Not |
| semantic_accuracy_v | 24.68 | 1.60×10⁻⁴ | Yes |
| semantic_accuracy_n_v | 20.44 | 0.001 | Yes |
| semantic_accuracy_c | 30.75 | 1.05×10⁻⁵ | Yes |
| semantic_richness_n | 59.1 | 1.86×10⁻¹¹ | Yes |
| semantic_clarity_n | 8.76 | 0.119 | Not |
| semantic_noise_n | 7.58 | 0.181 | Not |

Table S23. Pairwise Comparisons of Linguistic Features Among Six Models Under Rewriting Task (Prompt C).

| Indicator | Model_1 | Model_2 | p_value (uncorrected) | p_value (Bonferroni) | Significant |
| --- | --- | --- | --- | --- | --- |
| lexical_richness | ChatGPT | Claude | 0.394 | 1 | No |
| lexical_richness | ChatGPT | deepseek | 0.31 | 1 | No |
| lexical_richness | ChatGPT | gemini | 0.882 | 1 | No |
| lexical_richness | ChatGPT | grok | 0.579 | 1 | No |
| lexical_richness | ChatGPT | qwen | 0.133 | 1 | No |
| lexical_richness | Claude | deepseek | 0.946 | 1 | No |
| lexical_richness | Claude | gemini | 0.114 | 1 | No |
| lexical_richness | Claude | grok | 0.072 | 1 | No |
| lexical_richness | Claude | qwen | 0.003 | 0.046 | Yes |
| lexical_richness | deepseek | gemini | 0.25 | 1 | No |
| lexical_richness | deepseek | grok | 0.044 | 0.655 | No |
| lexical_richness | deepseek | qwen | 0.01 | 0.147 | No |
| lexical_richness | gemini | grok | 0.441 | 1 | No |
| lexical_richness | gemini | qwen | 0.457 | 1 | No |
| lexical_richness | grok | qwen | 0.108 | 1 | No |
| syntactic_richness | ChatGPT | Claude | 0.004 | 0.06 | No |
| syntactic_richness | ChatGPT | deepseek | 0.001 | 0.018 | Yes |
| syntactic_richness | ChatGPT | gemini | 0.047 | 0.702 | No |
| syntactic_richness | ChatGPT | grok | 0.001 | 0.021 | Yes |
| syntactic_richness | ChatGPT | qwen | 0.004 | 0.064 | No |
| syntactic_richness | Claude | deepseek | 0.441 | 1 | No |
| syntactic_richness | Claude | gemini | 0.164 | 1 | No |
| syntactic_richness | Claude | grok | 0.49 | 1 | No |
| syntactic_richness | Claude | qwen | 0 | 0.003 | Yes |
| syntactic_richness | deepseek | gemini | 0.068 | 1 | No |
| syntactic_richness | deepseek | grok | 0.776 | 1 | No |
| syntactic_richness | deepseek | qwen | 1.66E-07 | 2.49E-06 | Yes |
| syntactic_richness | gemini | grok | 0.024 | 0.366 | No |
| syntactic_richness | gemini | qwen | 0 | 0.001 | Yes |
| syntactic_richness | grok | qwen | 3.23E-05 | 4.85E-04 | Yes |
| semantic_accuracy_n | — | — | — | — | Not applicable (KW No) |
| semantic_accuracy_v | ChatGPT | Claude | 0.615 | 1 | No |
| semantic_accuracy_v | ChatGPT | deepseek | 0.473 | 1 | No |
| semantic_accuracy_v | ChatGPT | gemini | 0.057 | 0.848 | No |
| semantic_accuracy_v | ChatGPT | grok | 0.006 | 0.09 | No |
| semantic_accuracy_v | ChatGPT | qwen | 0 | 0.006 | Yes |
| semantic_accuracy_v | Claude | deepseek | 0.881 | 1 | No |
| semantic_accuracy_v | Claude | gemini | 0.091 | 1 | No |
| semantic_accuracy_v | Claude | grok | 0.014 | 0.206 | No |
| semantic_accuracy_v | Claude | qwen | 0.001 | 0.015 | Yes |
| semantic_accuracy_v | deepseek | gemini | 0.18 | 1 | No |
| semantic_accuracy_v | deepseek | grok | 0.032 | 0.473 | No |
| semantic_accuracy_v | deepseek | qwen | 0.004 | 0.064 | No |
| semantic_accuracy_v | gemini | grok | 0.06 | 0.895 | No |
| semantic_accuracy_v | gemini | qwen | 0.076 | 1 | No |
| semantic_accuracy_v | grok | qwen | 0.838 | 1 | No |
| semantic_accuracy_n_v | ChatGPT | Claude | 0.352 | 1 | No |
| semantic_accuracy_n_v | ChatGPT | deepseek | 0.776 | 1 | No |
| semantic_accuracy_n_v | ChatGPT | gemini | 0.068 | 1 | No |
| semantic_accuracy_n_v | ChatGPT | grok | 0.009 | 0.136 | No |
| semantic_accuracy_n_v | ChatGPT | qwen | 0.001 | 0.015 | Yes |
| semantic_accuracy_n_v | Claude | deepseek | 0.559 | 1 | No |
| semantic_accuracy_n_v | Claude | gemini | 0.024 | 0.366 | No |
| semantic_accuracy_n_v | Claude | grok | 0.127 | 1 | No |
| semantic_accuracy_n_v | Claude | qwen | 0 | 0.003 | Yes |
| semantic_accuracy_n_v | deepseek | gemini | 0.035 | 0.528 | No |
| semantic_accuracy_n_v | deepseek | grok | 0.047 | 0.702 | No |
| semantic_accuracy_n_v | deepseek | qwen | 0.007 | 0.101 | No |
| semantic_accuracy_n_v | gemini | grok | 0.324 | 1 | No |
| semantic_accuracy_n_v | gemini | qwen | 0.338 | 1 | No |
| semantic_accuracy_n_v | grok | qwen | 0.903 | 1 | No |
| semantic_accuracy_c | ChatGPT | Claude | 0.86 | 1 | No |
| semantic_accuracy_c | ChatGPT | deepseek | 0.715 | 1 | No |
| semantic_accuracy_c | ChatGPT | gemini | 0.047 | 0.702 | No |
| semantic_accuracy_c | ChatGPT | grok | 0.014 | 0.206 | No |
| semantic_accuracy_c | ChatGPT | qwen | 0 | 0.002 | Yes |
| semantic_accuracy_c | Claude | deepseek | 0.547 | 1 | No |
| semantic_accuracy_c | Claude | gemini | 0.019 | 0.284 | No |
| semantic_accuracy_c | Claude | grok | 0.006 | 0.09 | No |
| semantic_accuracy_c | Claude | qwen | 0 | 0.005 | Yes |
| semantic_accuracy_c | deepseek | gemini | 0.057 | 0.848 | No |
| semantic_accuracy_c | deepseek | grok | 0.021 | 0.311 | No |
| semantic_accuracy_c | deepseek | qwen | 0.003 | 0.044 | Yes |
| semantic_accuracy_c | gemini | grok | 0.285 | 1 | No |
| semantic_accuracy_c | gemini | qwen | 0.41 | 1 | No |
| semantic_accuracy_c | grok | qwen | 0.903 | 1 | No |
| semantic_richness_n | ChatGPT | Claude | 0.01 | 0.147 | No |
| semantic_richness_n | ChatGPT | deepseek | 3.53E-05 | 5.29E-04 | Yes |
| semantic_richness_n | ChatGPT | gemini | 1.06E-04 | 0.002 | Yes |
| semantic_richness_n | ChatGPT | grok | 9.75E-06 | 1.46E-04 | Yes |
| semantic_richness_n | ChatGPT | qwen | 0.18 | 1 | No |
| semantic_richness_n | Claude | deepseek | 0.007 | 0.101 | No |
| semantic_richness_n | Claude | gemini | 0.05 | 0.748 | No |
| semantic_richness_n | Claude | grok | 0.003 | 0.048 | Yes |
| semantic_richness_n | Claude | qwen | 0.001 | 0.015 | Yes |
| semantic_richness_n | deepseek | gemini | 0.441 | 1 | No |
| semantic_richness_n | deepseek | grok | 0.776 | 1 | No |
| semantic_richness_n | deepseek | qwen | 6.80E-08 | 1.02E-06 | Yes |
| semantic_richness_n | gemini | grok | 0.573 | 1 | No |
| semantic_richness_n | gemini | qwen | 4.30E-04 | 0.006 | Yes |
| semantic_richness_n | grok | qwen | 6.80E-08 | 1.02E-06 | Yes |
| semantic_clarity_n | — | — | — | — | Not applicable (KW No) |
| semantic_noise_n | — | — | — | — | Not applicable (KW No) |

Table S24. Impact of Prompt Strategy on Linguistic Features (Prompt A vs. Prompt B).

| Model | Indicator | U_statistic | p_value | Signifiacnt |
| --- | --- | --- | --- | --- |
| ChatGPT | lexical_richness | 400 | 6.80E-08 | Yes |
| ChatGPT | syntactic_richness | 144 | 0.1333 | No |
| ChatGPT | semantic_accuracy_n | 61 | 0.0002 | Yes |
| ChatGPT | semantic_accuracy_v | 0 | 6.80E-08 | Yes |
| ChatGPT | semantic_accuracy_n_v | 0 | 6.80E-08 | Yes |
| ChatGPT | semantic_accuracy_c | 0 | 6.80E-08 | Yes |
| ChatGPT | semantic_richness_n | 399 | 7.90E-08 | Yes |
| ChatGPT | semantic_clarity_n | 0 | 6.80E-08 | Yes |
| ChatGPT | semantic_noise_n | 261 | 0.1017 | No |
| Claude | lexical_richness | 400 | 6.80E-08 | Yes |
| Claude | syntactic_richness | 240 | 0.2853 | No |
| Claude | semantic_accuracy_n | 39 | 1.41E-05 | Yes |
| Claude | semantic_accuracy_v | 0 | 6.80E-08 | Yes |
| Claude | semantic_accuracy_n_v | 0 | 6.80E-08 | Yes |
| Claude | semantic_accuracy_c | 0 | 6.80E-08 | Yes |
| Claude | semantic_richness_n | 400 | 6.80E-08 | Yes |
| Claude | semantic_clarity_n | 193 | 0.8604 | No |
| Claude | semantic_noise_n | 389 | 3.42E-07 | Yes |
| deepseek | lexical_richness | 389 | 3.42E-07 | Yes |
| deepseek | syntactic_richness | 11 | 3.42E-07 | Yes |
| deepseek | semantic_accuracy_n | 149 | 0.1719 | No |
| deepseek | semantic_accuracy_v | 0 | 6.80E-08 | Yes |
| deepseek | semantic_accuracy_n_v | 0 | 6.80E-08 | Yes |
| deepseek | semantic_accuracy_c | 0 | 6.80E-08 | Yes |
| deepseek | semantic_richness_n | 398 | 9.17E-08 | Yes |
| deepseek | semantic_clarity_n | 104 | 0.0098 | Yes |
| deepseek | semantic_noise_n | 275 | 0.0439 | Yes |
| gemini | lexical_richness | 398.5 | 8.50E-08 | Yes |
| gemini | syntactic_richness | 234.5 | 0.3577 | No |
| gemini | semantic_accuracy_n | 41.5 | 1.92E-05 | Yes |
| gemini | semantic_accuracy_v | 11.5 | 3.66E-07 | Yes |
| gemini | semantic_accuracy_n_v | 3.5 | 1.15E-07 | Yes |
| gemini | semantic_accuracy_c | 4.5 | 1.33E-07 | Yes |
| gemini | semantic_richness_n | 389 | 3.40E-07 | Yes |
| gemini | semantic_clarity_n | 177.5 | 0.5518 | No |
| gemini | semantic_noise_n | 363.5 | 1.04E-05 | Yes |
| grok | lexical_richness | 400 | 6.80E-08 | Yes |
| grok | syntactic_richness | 114 | 0.0207 | Yes |
| grok | semantic_accuracy_n | 82 | 0.0015 | Yes |
| grok | semantic_accuracy_v | 68 | 0.0004 | Yes |
| grok | semantic_accuracy_n_v | 19 | 1.05E-06 | Yes |
| grok | semantic_accuracy_c | 16 | 6.92E-07 | Yes |
| grok | semantic_richness_n | 376 | 2.06E-06 | Yes |
| grok | semantic_clarity_n | 35 | 8.60E-06 | Yes |
| grok | semantic_noise_n | 320 | 0.0012 | Yes |
| qwen | lexical_richness | 400 | 6.80E-08 | Yes |
| qwen | syntactic_richness | 346 | 8.29E-05 | Yes |
| qwen | semantic_accuracy_n | 52 | 6.61E-05 | Yes |
| qwen | semantic_accuracy_v | 0 | 6.80E-08 | Yes |
| qwen | semantic_accuracy_n_v | 0 | 6.80E-08 | Yes |
| qwen | semantic_accuracy_c | 0 | 6.80E-08 | Yes |
| qwen | semantic_richness_n | 400 | 6.80E-08 | Yes |
| qwen | semantic_clarity_n | 58 | 0.0001 | Yes |
| qwen | semantic_noise_n | 396 | 1.23E-07 | Yes |

Table S25. Comparison of Linguistic Features Between AI-Rewritten Texts and Original Baseline Texts (Prompt C vs. Original).

| Model | Indicator | Statistic | p_value_formatted | Signifiacnt |
| --- | --- | --- | --- | --- |
| ChatGPT | lexical_richness | 46 | 0.0266 | Yes |
| ChatGPT | syntactic_richness | 69 | 0.1893 | No |
| ChatGPT | semantic_accuracy_n | 38 | 0.0107 | Yes |
| ChatGPT | semantic_accuracy_v | 3 | 9.54E-06 | Yes |
| ChatGPT | semantic_accuracy_n_v | 0 | 1.91E-06 | Yes |
| ChatGPT | semantic_accuracy_c | 0 | 1.91E-06 | Yes |
| ChatGPT | semantic_richness_n | 0 | 1.91E-06 | Yes |
| ChatGPT | semantic_clarity_n | 37 | 0.0094 | Yes |
| ChatGPT | semantic_noise_n | 92 | 0.6477 | No |
| Claude | lexical_richness | 31 | 0.0042 | Yes |
| Claude | syntactic_richness | 54 | 0.0583 | No |
| Claude | semantic_accuracy_n | 13 | 0.0002 | Yes |
| Claude | semantic_accuracy_v | 1 | 3.81E-06 | Yes |
| Claude | semantic_accuracy_n_v | 0 | 1.91E-06 | Yes |
| Claude | semantic_accuracy_c | 0 | 1.91E-06 | Yes |
| Claude | semantic_richness_n | 0 | 1.91E-06 | Yes |
| Claude | semantic_clarity_n | 49 | 0.0362 | Yes |
| Claude | semantic_noise_n | 58 | 0.0826 | No |
| deepseek | lexical_richness | 31 | 0.0042 | Yes |
| deepseek | syntactic_richness | 51 | 0.0441 | Yes |
| deepseek | semantic_accuracy_n | 35 | 0.0073 | Yes |
| deepseek | semantic_accuracy_v | 1 | 3.81E-06 | Yes |
| deepseek | semantic_accuracy_n_v | 0 | 1.91E-06 | Yes |
| deepseek | semantic_accuracy_c | 0 | 1.91E-06 | Yes |
| deepseek | semantic_richness_n | 0 | 1.91E-06 | Yes |
| deepseek | semantic_clarity_n | 24 | 0.0014 | Yes |
| deepseek | semantic_noise_n | 87 | 0.5217 | No |
| gemini | lexical_richness | 23 | 0.0012 | Yes |
| gemini | syntactic_richness | 24 | 0.0014 | Yes |
| gemini | semantic_accuracy_n | 22 | 0.001 | Yes |
| gemini | semantic_accuracy_v | 0 | 1.91E-06 | Yes |
| gemini | semantic_accuracy_n_v | 0 | 1.91E-06 | Yes |
| gemini | semantic_accuracy_c | 0 | 1.91E-06 | Yes |
| gemini | semantic_richness_n | 0 | 1.91E-06 | Yes |
| gemini | semantic_clarity_n | 56 | 0.0696 | No |
| gemini | semantic_noise_n | 63 | 0.1231 | No |
| grok | lexical_richness | 30 | 0.0037 | Yes |
| grok | syntactic_richness | 81 | 0.3884 | No |
| grok | semantic_accuracy_n | 36 | 0.0083 | Yes |
| grok | semantic_accuracy_v | 0 | 1.91E-06 | Yes |
| grok | semantic_accuracy_n_v | 0 | 1.91E-06 | Yes |
| grok | semantic_accuracy_c | 0 | 1.91E-06 | Yes |
| grok | semantic_richness_n | 0 | 1.91E-06 | Yes |
| grok | semantic_clarity_n | 45 | 0.024 | Yes |
| grok | semantic_noise_n | 51 | 0.0441 | Yes |
| qwen | lexical_richness | 49 | 0.0362 | Yes |
| qwen | syntactic_richness | 87 | 0.5217 | No |
| qwen | semantic_accuracy_n | 17 | 0.0004 | Yes |
| qwen | semantic_accuracy_v | 0 | 1.91E-06 | Yes |
| qwen | semantic_accuracy_n_v | 0 | 1.91E-06 | Yes |
| qwen | semantic_accuracy_c | 0 | 1.91E-06 | Yes |
| qwen | semantic_richness_n | 0 | 1.91E-06 | Yes |
| qwen | semantic_clarity_n | 35 | 0.0073 | Yes |
| qwen | semantic_noise_n | 70 | 0.2024 | No |
